# Global Identification of Solid Waste Methane Super Emitters Using Hyperspectral Satellites

**DOI:** 10.1021/acs.est.4c14196

**Published:** 2025-08-19

**Authors:** Xin Zhang, Joannes D. Maasakkers, Javier Roger, Luis Guanter, Shubham Sharma, Srijana Lama, Paul Tol, Daniel J. Varon, Daniel H. Cusworth, Katherine Howell, Andrew K. Thorpe, Philip G. Brodrick, Ilse Aben

**Affiliations:** † SRON Space Research Organisation Netherlands, Leiden 2333 CA, The Netherlands; ‡ Research Institute of Water and Environmental Engineering, Universitat Politècnica de València, Valencia 46022, Spain; § Environmental Defense Fund, Amsterdam 1083 HN, The Netherlands; ∥ School of Engineering and Applied Sciences, 1812 Harvard University, Cambridge, Massachusetts 02138, United States; ⊥ Carbon Mapper, Pasadena, California 91105, United States; # Jet Propulsion Laboratory, California Institute of Technology, Pasadena, California 91109, United States; ¶ Department of Earth Sciences, Vrije Universiteit Amsterdam, Amsterdam 1081 HV, The Netherlands

**Keywords:** methane, hyperspectral, landfill, satellite, remote sensing

## Abstract

Solid waste is the third largest source of anthropogenic methane, and mitigating emissions is crucial for addressing climate change. We combine three high-resolution (30–60 m) hyperspectral satellite imagers (EMIT, EnMAP, and PRISMA) to quantify emissions from 38 strongly emitting disposal sites across worldwide urban methane hotspots. The imagers give consistent emission estimates, with EMIT and EnMAP having better sensitivity than PRISMA. Total observed emissions add up to 230 ± 15 t h^–1^, representing 5% of reported global solid waste emissions. Our estimates exceed the facility-level Climate TRACE inventory by a factor of 1.8, while we only detect emissions from 9 of the inventory’s 20 highest-emitting sites, highlighting the importance of facility-level information. Furthermore, multimonth observations reveal emission patterns potentially linked to facility operations. We estimate that these instruments could detect 35–60% of global landfill emissions, critically expanding on satellite instruments designed for methane and supporting emission mitigation.

## Graphical abstract

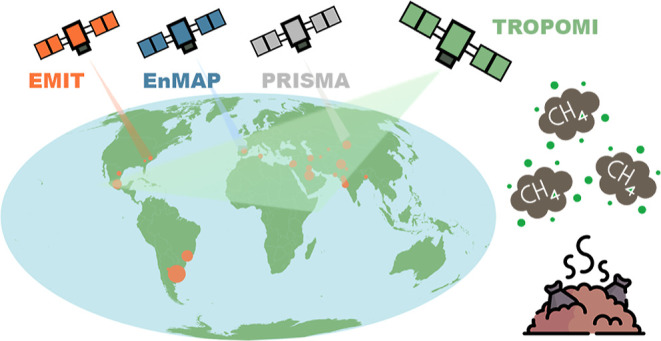

## Introduction

Methane is a potent greenhouse gas with a global warming potential 27–30 times higher than that of carbon dioxide over a 100 year time scale.^1^ Its relatively short atmospheric lifetime of about a decade makes reducing methane emissions critical for mitigating near-term global warming. Anthropogenic activities account for ∼60% of global methane emissions, with waste treatment as the third largest source (19%) after agriculture and fossil fuel exploitation.^2^ Moreover, the global waste generation could increase by ∼60% from 2016 to 2050.^3^ Waste methane emission reductions have become a priority for global climate action, as exemplified by the “Declaration on Reducing Methane from Organic Waste” declaration introduced at the 29th UN Climate Change Conference (COP29).^4^ In this declaration, countries responsible for over 50% of organic waste methane emissions committed to including reduction strategies in their climate plans. Several countries already announced specific plans, and the Lowering Organic Waste Methane (LOW-Methane) initiative is focused on reducing annual global waste methane emissions by one million metric tonnes a year by 2030 and unlocking 10 billion dollars in funding to achieve this goal.^5^

However, accurately quantifying landfill methane emissions remains challenging, with substantial uncertainties in both site-specific and global estimates.^6–9^ While traditional approaches rely on modeling and limited aircraft measurements and can only cover a limited number of sites,^6,10–13^ space-borne monitoring offers a way to improve emission quantification across the world using consistent methodology.^14^ A 2022 study^15^ demonstrated the application of GHGSat observations to quantify emissions from four landfills, including one in Buenos Aires that contributed 50% of the city’s methane emissions. However, facility-scale coverage by satellites designed to observe methane is currently limited. Here, we therefore evaluate the potential of using alternative imaging spectrometers to extend that coverage and quantify emissions from individual landfills.

The TROPOspheric Monitoring Instrument (TROPOMI)^16,17^ has been used for monitoring regional methane emissions^18,19^ and detecting urban superemitters.^15,20^ However, its spatial resolution (5.5 × 7 km^2^ at nadir) typically cannot separate landfill emissions from other city emissions.^15^ Currently, the only operational spaceborne instruments specifically designed to measure methane at facility-level are the commercial satellites from the GHGSat constellation.^21,22^ A small fraction of the GHGSat data is publicly available, and individual observations only cover an area of ∼12 × 15 km^2^. Recent studies highlight the use of public multispectral^23–25^ and hyperspectral imagers (HSIs)^26–28^ for detecting large point sources, primarily from the oil/gas industry. HSIs, similar to the Airborne Visible InfraRed Imaging Spectrometer - Next Generation (AVIRIS-NG),^8,29^ are not designed for methane detection but offer relatively high methane sensitivity through hundreds of narrow spectral bands. Starting with PRecursore IperSpettrale della Missione Applicativa (PRISMA),^30,31^ HSIs have been verified to be capable of detecting plumes down to 300–500 kg h^–1^ in favorable conditions such as bright homogeneous desert scenes,^32,33^ outperforming multispectral sensors such as Sentinel-2.^23–25^ Thus, HSIs are particularly promising for detecting landfill methane emissions, which are more diffused than those from oil/gas operations and occur over a more complex terrain.

Previous studies have demonstrated the potential of HSIs in detecting landfill methane emissions. The Environmental Mapping and Analysis Program (EnMAP)^34,35^ has been used to detect emissions from the Ghazipur and Okhla landfills in Delhi,^28^ while Earth Surface Mineral Dust Source Investigation (EMIT)^34,35^ has been used to detect emissions from 11 different landfills around the world.^27^ To assist in mitigating global landfills, it is crucial to construct a comprehensive global landfill emission data set. Here, we integrate TROPOMI and three HSIs (EMIT, EnMAP, and PRISMA) to identify, quantify, and monitor high-emission landfills worldwide. As part of the analysis, we compare the performance of all HSIs and examine the impact of wind speed uncertainty on emission quantification. We also compare our results to existing emission inventories. Our analysis assesses hyperspectral imaging’s potential to monitor global landfill methane, expanding upon current satellite capabilities designed for methane observation.

## Results

### Landfill Methane Hot Spots

Figure 1 shows an overview of urban and landfill methane hot spots detected by TROPOMI and HSIs, along with examples of typical methane plumes observed by HSIs. Using 2020–2023 TROPOMI data, we identified persistent global urban methane hot spots based on plume detections and analysis of long-term averages (see the Methods section).^15,20^ Among all hot spots, 58 are potentially associated with landfill emissions, given their source locations, although they may also include contributions from other urban sources. We evaluate 46 landfills within these TROPOMI hot spots using EMIT and EnMAP, while the remaining 12 lack observations. PRISMA has clear-sky observations for 49 landfills (Figure S8) but only detects plumes from 4 due to its lower methane sensitivity, caused by the lower signal-to-noise ratio (SNR) and spectral resolution (see the Methods section).

**Figure 1:**
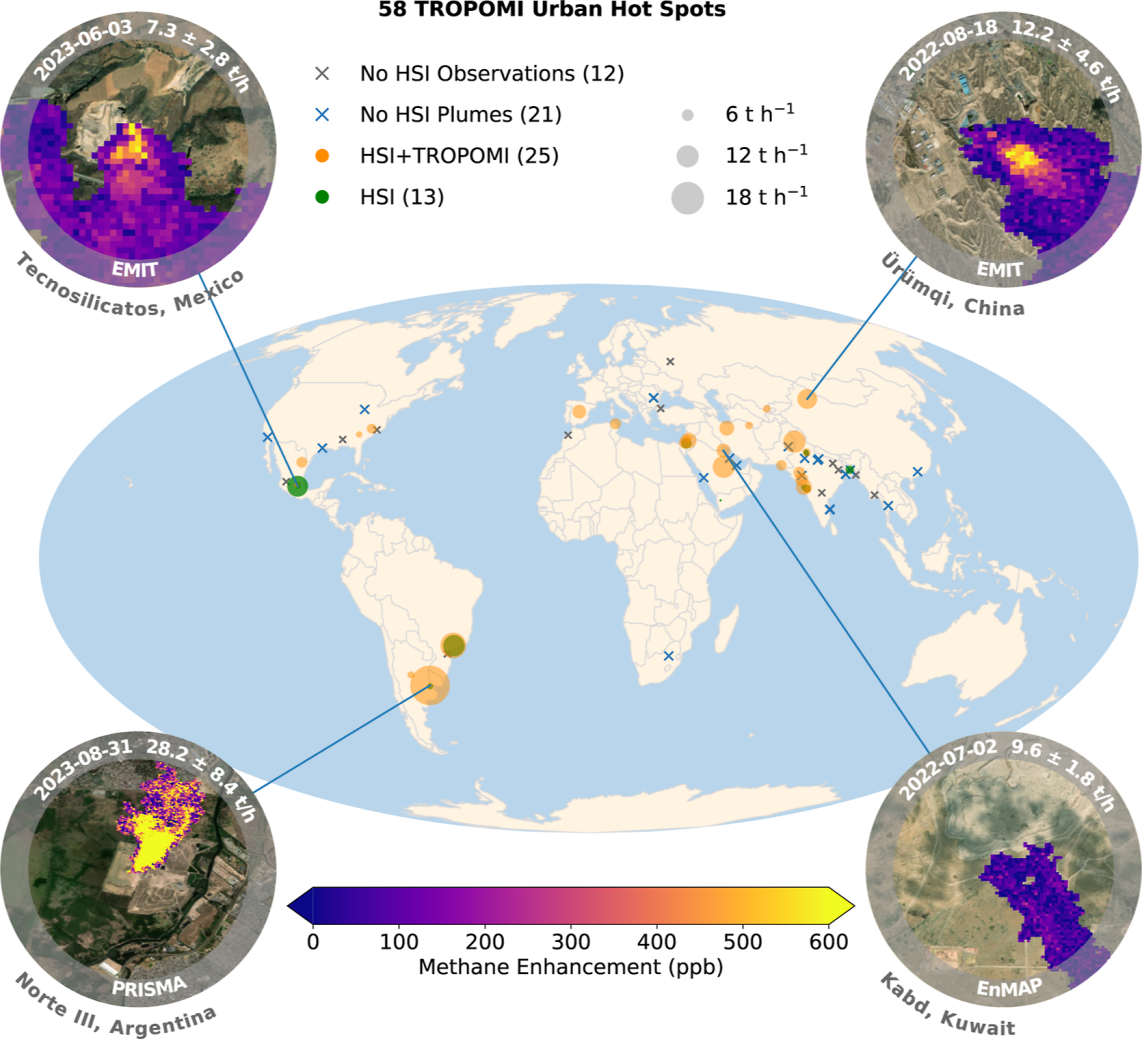
Urban hot spots detected by TROPOMI (2020–2023) and landfill emissions detected at those hot spots using hyperspectral imagers (HSIs) including EMIT, EnMAP, and PRISMA. Gray crosses indicate TROPOMI hot spots without clear-sky HSI data, blue crosses show hot spots with clear-sky HSI observations without detected plumes, orange circles show TROPOMI hot spots with HSI plumes, and green circles indicate plumes detected by HSIs slightly away from the TROPOMI hot spots. The “No HSI Observations” group excludes PRISMA due to its lower methane sensitivity. The insets show typical landfill plumes with detection date, emission rate, uncertainty, landfill/country name, and instrument. Background imagery reproduced with permission as granted on Esri’s website for noncommercial scholarly use.^36^ Figure S7 shows a zoomed-in view of landfill emissions across India.

Overall, the HSI data reveal detectable plumes from 38 landfills: 25 within 15 km of TROPOMI hot spots and 13 at nearby locations (Figure 1). EMIT, with its wider scene coverage, observes all 38 landfills in clear-sky conditions and detects plumes from 36 (Figure S8). EnMAP shows a comparable capability, detecting plumes from 16 out of 18 observed landfills, while PRISMA, due to its lower sensitivity, detects plumes only at 4 out of 32 observed sites. Among the 38 landfills with detected plumes, 29 are observed at least twice, with 10 having 8–14 plume detections, facilitating emission time series analysis (see the Emission Variations section). The total number of plumes detected by each HSI is as follows: EMIT observes 132 plumes, EnMAP 38, and PRISMA 10 (Figure S9).

This highlights the potential of EMIT and EnMAP in identifying landfill emission sources, whereas PRISMA is constrained by a higher detection threshold. When calculating mean emission rates, we use different approaches for each instrument. When no plumes are detected during clear-sky overpasses by EnMAP and EMIT (5 cases total; see Table S1), we make the conservative assumption of zero emissions. If we would instead exclude these cases from the estimates, the resulting mean emission rates would remain within our estimated uncertainties. In the case of PRISMA, owing to its lower sensitivity, we only include instances where plumes are detected in our emission rate calculations.

### Landfill Methane Emission Rates

A commonly used data-driven approach for methane retrieval from HSIs involves a matched filter algorithm that maximizes the signal-to-background ratio by identifying pixels exhibiting the strongest correlation with methane’s absorption spectrum. We improve the traditional matched filter to retrieve methane enhancements using Level 1 radiance data and to estimate emission rates through the integrated mass enhancement (IME) method, specifically calibrated for each instrument (see the Methods section). The reported uncertainties include contributions from wind speed error, retrieval random error, and IME calibration error (Supporting Information Section S1). We validate our methodology using two controlled releases (Supporting Information Section S2), one for PRISMA (October 21, 2021) and one for EnMAP (November 16, 2022). Both controlled releases show that our satellite estimates agree with the controlled flow rates within their uncertainties (Figure S2). While these validations are performed using point-source controlled releases, we expect that controlled releases simulating more dispersed emissions from landfills will become available in the near-future. While the overpasses for different HSIs typically vary in timing over the same landfill, the average magnitudes of emission rates between EnMAP and EMIT are consistent (slope = 1.21 ± 0.17, *r* = 0.84, Figure S10a). We therefore use data from both instruments together for the remainder of this study. PRISMA’s emission rate estimates for two landfills are consistent with those from EMIT and EnMAP in the same year (Figure S10b).

Figure 2 shows our methane emission rates for 38 landfills found near 25 TROPOMI-identified hot spots across 17 countries, with the lowest rate being ∼1 t h^–1^. These sites are among the world’s largest sanitary landfills and dumpsites.^9^ The sum of mean emission rates across sites is 230 ± 15 t h^–1^, with most of the observed high-emission landfills located at hot spots in India, Argentina, Brazil, and Mexico. India stands out with the highest total of 41.4 ± 5.0 t h^–1^ from 10 landfills. Argentina follows at 28.1 ± 6.6 t h^–1^, primarily driven by the Norte III landfill in Buenos Aires, showing the highest emission rate among all observed landfills at 22.0 ± 6.4 t h^–1^. Brazil has a similar emission of 25.6 ± 6.3 t h^–1^, with the Caieiras (14.0 ± 4.8 t h^–1^) and Pedreira (11.5 ± 4.0 t h^–1^) landfills in Sao Paulo strongly contributing to this total. These three large-emitting landfills in Buenos Aires and Sao Paulo account for 20% of the total quantified landfill methane emissions. Mexico ranks fourth at 23.7 ± 5.3 t h^–1^, half of which comes from the Tecnosilicatos landfill in Mexico City.

**Figure 2:**
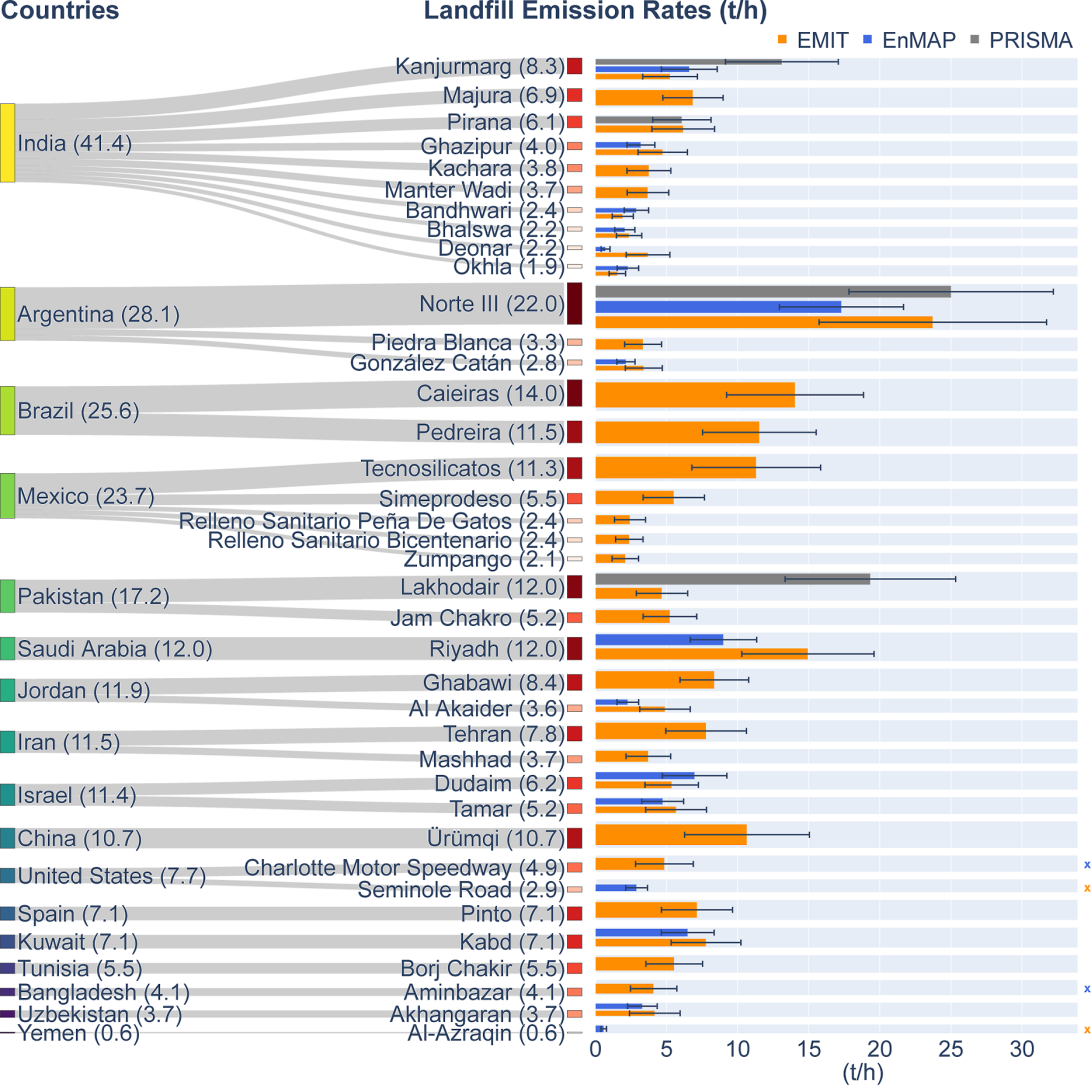
Sankey plot for the landfill emissions estimated using hyperspectral imagers (HSIs). Box heights are proportional to emission rates (t h^–1^), with values in brackets. Colored bars show estimates from different instruments, with uncertainties in black. Crosses on the right indicate EMIT or EnMAP overpasses without detected methane plumes. Nondetections with PRISMA are not depicted, given PRISMA’s lower sensitivity. More details are given in Tables S3 and S2.

Among the remaining 13 countries, each with only 1 to 2 observed landfills, six have a total emission rate ranging from 10 to 17 t h^–1^. This can be attributed to the presence of large emitting landfills, such as the Lakhodair landfill (12.0 ± 4.2 t h^–1^) in Pakistan, the Riyadh landfill (12.0 ± 3.4 t h^–1^) in Saudi Arabia, the Ürümqi landfill (10.7 ± 4.4 t h^–1^) in China, the Ghabawi landfill (8.4 ± 2.4 t h^–1^) in Jordan, and the Tehran landfill (7.8 ± 2.8 t h^–1^) in Iran. The cumulative distribution reveals that for this set of 38 landfills, the top 20% highest emitters contribute 46% of the inferred total emission (Figure S11a). This highlights the importance of detecting and mitigating high methane-emitting landfills. Due to variations in background noise levels, wind speed, and potential methane emission variability, landfill methane plumes are sometimes detected by one HSI and missed by another (crosses in Figure 2). This emphasizes the value of combining multiple HSIs to monitor landfill emissions. However, in most cases, both EnMAP and EMIT detect emissions from specific landfills, thereby increasing the observation opportunities for the emission of landfill emissions. For cases with a single detected plume (Figure S9), estimates may be affected by potential offsets. Future studies with more data will be crucial for refining these constraints.

### Comparison with Observations and Inventories

First, we compare our HSI estimates with recent satellite, aircraft, and ground-based observations (Figure 3a).^15,27,37,38^ For eight of the observed landfills, there are estimates from earlier studies. Our HSI results show good agreement with these estimates (slope = 1.31 ± 0.14, *r* = 0.97, Figure 3a), though the number of data points is limited (Table S4). We then compare our facility-level methane emission estimates with the Climate Tracking Real-time Atmospheric Carbon Emissions (Climate TRACE) data set, which models emissions using multiple waste data sets (see the Methods section). To compare them on the same time scale, we convert the yearly Climate TRACE emissions into hourly emissions, assuming constant emission rates. We find that the Climate TRACE data set generally underestimates landfill emissions compared to HSI for the 26 landfills with overlapping estimates (Figure 3a and Table S5). Based on the HSI measurements, total methane emissions (141 ± 11 t h^–1^) from these landfills are 1.8 times higher than the estimates in the Climate TRACE inventory. Some of the data used in the Climate Trace inventory may be outdated. For example, the Norte III landfill data from the 2013 Waste Atlas report emissions of 3.3 t h^–1^, significantly lower than our estimate of 22.0 ± 6.4 t h^–1^. Considering only the 2021 and 2022 Climate Trace data for 15 landfills, our estimates are only 1.3 times higher. However, upon comparison of individual facilities, the median ratio between our estimates and the Climate TRACE data is still 4.7, exceeding the 1.6 ratio found in comparisons with previous studies. Therefore, the differences appear to be related not only to up-to-date information on landfill activities but also to appropriate emission factors representative of operations at different landfills.

**Figure 3:**
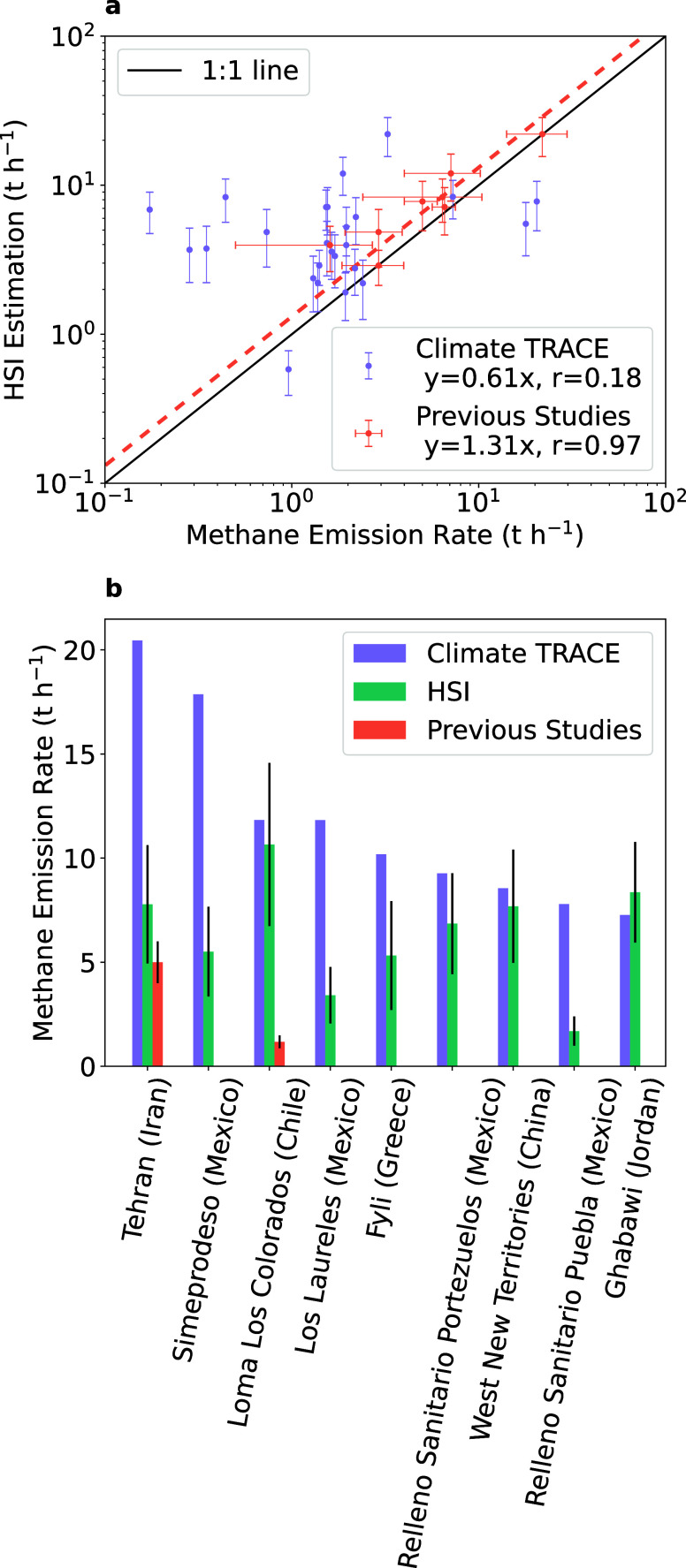
Comparison of methane emission rates from hyperspectral imager (HSI) observations, the Climate TRACE inventory, and observational estimates from satellite and aircraft studies for (a) landfills mapped in Figure 1, and (b) the top 20 methane-emitting landfills in the Climate TRACE data set (see Tables S4–S6 for details). The regression coefficients are calculated using orthogonal distance regression. The Pearson correlation coefficients are 0.18 between HSI and Climate TRACE and 0.97 between HSI and previous studies.

In addition to the landfills at hot spots, we then focus on Climate TRACE’s top 20 highest emitting landfills (Figure 3b and Table S6). HSIs overpass all 20 landfills but only detect plumes from 9 still-active landfills, while the remaining 11 appear inactive based on vegetation covering the landfill as seen in Sentinel-2 imagery (Figure S12). For example, the Naameh landfill in Lebanon was closed in 2015 ^39^ and appears vegetated in Sentinel-2 imagery. However, Climate TRACE has not updated this information and continues to list it as the third-largest active landfill emitter in the world (Table S7). Among nine active landfills, our estimates are consistent with Climate TRACE for four but are 48 ∼71% lower for the other five. For two of these landfills (Tehran and Loma Los Colorados), additional observational estimates are available in the literature. Our estimate for the Tehran landfill agrees with an earlier EMIT analysis.^27^ However, four AVIRIS-NG observations of the Loma Los Colorados landfill in January and February 2023 reported emissions of 1.2 ± 0.3 t h^–1^,^38^ which is 89% lower than our EMIT-based estimate for January and 90% lower than the Climate TRACE estimate. These results show that differences between facility-level observations and bottom-up estimates can go both ways and that there may be substantial temporal variability in emissions. Some variability may also be due to differences in quantification algorithms applied to remote sensing data sets. Using the same EMIT observations, we compare methane emissions across 36 landfills using Carbon Mapper’s IME-fetch method (Supporting Information Section S4). We find that some significant variability can be traced to quantification uncertainties, particularly in plume masking. This variability can be reproduced by using large-eddy simulations. Despite these variations, the overall emission results remain consistent across quantification algorithms for most landfills in this study.

In addition to facility-level comparisons, we evaluate how our HSI estimates compare to solid waste methane emission inventories at the city scale from the Waste Methane Assessment Platform (WasteMAP). Of the 15 cities included in both the WasteMAP platform and our analysis, accounting for uncertainties, only two have emissions in WasteMAP higher than those in our summed HSI landfill estimates (Figure S13a and Table S7). HSI emissions from the Pinto (Spain), Simeprodeso (Mexico), and Borj Chakir (Tunisia) landfills alone are 16 ∼27 times higher than total city emissions for Madrid, Monterrey, and Tunis, respectively. The mean ratio of our HSI-derived landfill emissions to city totals is 6.3. One reason for this high discrepancy may be that these landfills service a larger area than the cities they are within. For example, while WasteMAP only uses the Tunis population of 0.7 million in its emission estimation of the Borj Chakir landfill, the site serves 38 municipalities across Tunisia, covering a total population of about 3 million.^40^ This discrepancy could partly explain why the WasteMAP emission estimate is 18 times lower than our satellite-based estimate (Table S7), suggesting that the landfill’s total emissions are more closely proportional to the total population serviced by the landfill rather than just the urban population of Tunis used in the WasteMAP calculation.

At the country level, Climate TRACE solid waste emissions generally exceed the sum of our HSI landfill emissions (Figure S13b and Table S8). This difference arises because HSI measurements typically only cover a small fraction of the landfills included in the Climate TRACE data, while Climate TRACE’s country-level inventory considers all solid waste emissions. However, Climate TRACE’s total facility-level emissions are 47% lower than HSI estimates in six countries, while the remaining countries show emissions that are either higher than or comparable to HSI estimates (Figure S13b). These findings highlight the importance of evaluating and improving emission inventories across scales using observations, particularly accounting for strongly emitting landfills that may be underestimated in current inventories.

### Emission Variations

The multiple overpasses of HSIs enable us to examine the spatial and temporal variations in emissions (Figure S14). Specifically, the Ghabawi landfill in Jordan has a total of 14 EMIT observations, with measurements taken every 1–2 months throughout 2023 (Figure 4). Between February and April 2023, the emission rate increased from 5.1 ± 1.7 t h^–1^ to 17.2 ± 4.3 t h^–1^. Then, it decreased to 3.9 ± 1.8 t h^–1^ in September, before increasing again to 9.3 ± 2.1 t h^–1^ in December.

**Figure 4:**
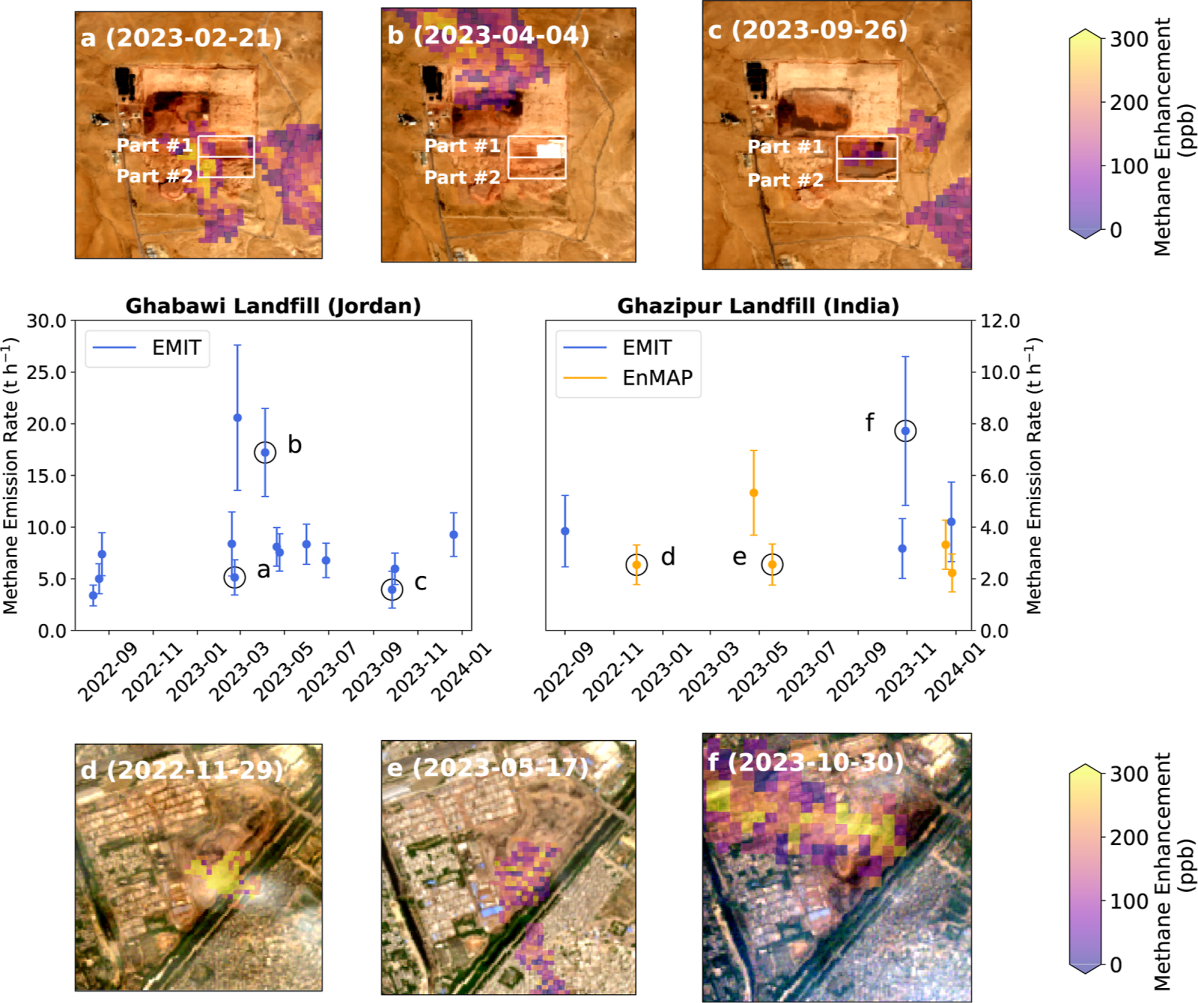
Time series of methane emissions from the Ghabawi (Jordan) and Ghazipur (India) landfills as derived using EMIT and EnMAP data. The complete Sentinel-2 RGB time series for 2023 are available as Movies S1 and S2. The points marked with letters (a–f) correspond to the insets labeled with matching letters in their upper left corners. (a–c) Methane plumes observed at the Ghabawi landfill shown over Sentinel-2 images^41^ captured within 3 days of the EMIT overpass: (a) February 21, 2023, (b) April 4, 2023, (c) September 26, 2023. The white rectangles highlight two sections in the newly constructed southern section. (d–f) Similar observations for the Ghazipur landfill: (d) November 29, 2022, (e) May 17, 2023, (f) October 30, 2023.

The variation in emission rates is not correlated with the wind speed magnitude, as indicated by Fisher’s combined probability test (*p* = 0.5). It is also seen when using an alternate wind product and quantification method to calculate emission rates (Supporting Information Section S1, Figures S15 and S16). We then tracked waste disposal activities using Sentinel-2 RGB images captured within 3 days of each EMIT overpass (Figure 4a–c). These images show a shift in the plume source location from the northern cell to a newly established southern cell. The southern cell is part of an engineered expansion at Al Ghabawi, Jordan’s largest landfill, which receives 2295 tons of municipal solid waste daily.^42^ The year-round Sentinel-2 images (Figure S17 and Movie S1) show that the construction process of the southern cell was divided into two phases: March to June (part #1, Figure 4b) and June to September (part #2, Figure 4c), while waste deposition in the cell began in August. This phased construction agrees with reporting that the first half of the southern cell was completed by late May 2023, while the full cell was operational by August 2023.^43^ Although the spike in methane emission rates coincides with the active construction of part #1 in April, the plume’s source is not located within this newly constructed area. Instead, it originates from waste deposited in earlier phases of the landfill (Figure 4b). These observations align with previous studies highlighting how variability in landfill emissions is heavily influenced by operational procedures, such as the choice of cover material or alterations in landfill infrastructure, alongside local weather conditions.^12,44^ Future engagement with landfill operators is necessary to provide deeper insights into the emission variations. Retrieval artifacts can also cause minor variations due to the confounding influence of the landfill’s surface materials in the methane retrieval spectral window (2100–2450 nm).

Given the sparse temporal sampling of landfills by individual HSI instruments, combining observations from all available HSI sensors is valuable for exploring emission time series. The Ghazipur landfill in Delhi, India, is an illustrative example (Figure 4d–f). Despite infrequent revisits, we find that the emission source shifted from the southern section to the northeast, corresponding to increasing activity in the northeastern section, as shown by the Sentinel-2 images (Figure S18 and Movie S2). The combined analysis of HSI data and satellite imagery demonstrates the capability to capture both spatial and temporal changes in landfill operations and associated methane emissions. When more HSI observations become available in the future, they will help us estimate baseline methane emissions more accurately and improve long-term projections of landfill methane emissions.

## Discussion

We analyzed global methane emissions from landfills by integrating observations from TROPOMI and HSIs. TROPOMI first identifies urban hot spots indicative of potentially large landfill methane emissions, which are then targeted by the analysis of HSIs. Our findings reveal differences with current landfill emission inventories, highlighting the critical need for observation-based updates to account for super-emitting sites. Furthermore, measurements from different HSIs can be used to monitor emissions over time at any specific site and enable exploration of emission variability resulting from operational procedures. This synergistic use of spaceborne sensors establishes a robust framework for the continuous global monitoring of landfill methane emissions. Given that 80% of landfill methane emissions could be mitigated through existing technological solutions such as gas capture,^45,46^ our spaceborne methane emission products can identify high-emitting sites to prioritize gas recovery infrastructure, pinpoint emissions within the site, improve baseline emission estimates in conjunction with emission modeling, and track emission reductions post-implementation.^5^

This study is limited to only the largest emitting hotspots due to TROPOMI’s ∼8 t h^–1^ detection threshold.^20^ The cumulative distribution of Climate TRACE emissions shows that 5% of global landfill methane emissions can be detected under this constraint (Figure S11b). While this study targets only 0.4% of landfills in the Climate TRACE data set, these sites account for ∼5% of their estimated global landfill emissions (36.8 Tg yr^–1^), a global total similar to the one from another independent inventory study (31.9 Tg yr^–1^).^45^ On the other hand, HSIs detect plumes only from the Tehran landfill among the Climate TRACE landfills emitting more than 8 t h^–1^, suggesting large facility-level differences.

While the empirical detection limits are 810 kg h^–1^ for EnMAP and 970 kg h^–1^ for EMIT (Supporting Information Section S5), this study’s lowest two observed emission rates are 900 and 1050 kg h^–1^, respectively. Considering the limited sample of 18 observed emission rates below 2 t h^–1^ and the spatially diffuse nature of landfill emissions, which makes them more challenging to detect, we estimate that HSIs have a detection threshold of 1–2 t h^–1^. At this threshold, 35–60% of solid waste emissions could be observable through global monitoring (Figure S11b). As spatial/temporal coverage of HSI observations is currently limited, expanding HSI monitoring to more sites by increasing landfill target coverage and implementing automated plume detection^47,48^ will enable more comprehensive top-down information. Moreover, additional facility-level data will soon become available from satellites designed to observe methane and carbon dioxide, including MethaneSAT (launched March 2024, 100 × 400 m^2^ resolution, 500 kg/h detection limit)^49,50^ and Carbon Mapper Coalition’s Tanager-1 hyperspectral satellite (launched August 2024, ∼35 m resolution, 90–180 kg/h detection limit).^51,52^ To support all these, further validation with controlled releases from landfill-like sources is needed, particularly over complex terrain. The growing constellation of methane-monitoring satellites—integrating TROPOMI and MethaneSAT’s wide coverage (200 × 200 km^2^) with high-resolution targeted observations—will improve our framework’s spatial–temporal emission estimates, especially for landfills emitting below 1 t h^–1^, and strengthen mitigation planning in collaboration with landfill operators. Further observations using aircraft or ground-based measurements could provide additional valuable data. Combining observational estimates with bottom-up modeling approaches offers the greatest value, enhancing model accuracy while preserving traceable emission inventories that can quantify the effectiveness of mitigation strategies implemented at landfill sites.

## Methods

### Hyperspectral Imagers

We combined three push-broom hyperspectral imagers (400–2500 nm) to detect global landfill methane emissions: EMIT,^34,35^ launched on July 14, 2022 and operating on the International Space Station (ISS); EnMAP,^53,54^ launched on April 1, 2022; and PRISMA,^30,31^ launched on March 22, 2019. EnMAP and PRISMA provide 30 m spatial resolution over 30 × 30 km^2^ scenes, while EMIT operates at 60 m resolution but covers a wider 80 km scene. EnMAP and PRISMA are in Sun-Synchronous Low Earth Orbits with equator crossing times of 11:00 and 10:30, respectively, while EMIT has a variable overpass time. At the strong methane absorption window (∼2300 nm), EMIT outperforms EnMAP and PRISMA with a SNR of ∼500 and a spectral resolution of 7.4 nm.^55^ In contrast, EnMAP’s SNR is twice that of PRISMA (∼180), and its spectral resolution is 2.7 nm finer than PRISMA’s 10 nm resolution.^28,56^

Given the substantial size of the hyperspectral data sets, we initially focus on urban hot spots detected by TROPOMI (https://methanedata.unep.org/) where the wind rotation technique is used to determine the source location within a few km.^15,20^ Then, we restricted our investigation to the surrounding area to determine whether the detected emissions originate from waste disposal sites or other sources and estimate their emission rates. Additionally, we analyze observations of the top 20 most emitting landfills from the Climate TRACE data set.

### Methane Enhancement Retrieval

We employ a linearized matched filter technique to retrieve methane enhancements (ΔXCH_4_) in parts per billion (ppb) from satellite observations. This approach has been successfully applied before to satellite and aircraft observations.^27,57–61^ The matched filter assumes a spectrally flat background and models the background radiance spectrum as a Gaussian distribution (N) with a mean vector μ and a covariance matrix **Σ**. The radiance spectrum (*L*) can be represented by two hypotheses: *H*_0_ for radiance without a methane plume and *H*_1_ with a plume present.^57^

1 $$H_{0}:L∼N\left(\mu ,\Sigma \right);H_{1}:L∼N\left(\mu +\Delta {XCH}_{4}t,\Sigma \right)$$

Here, ***t*** represents the target signature, the product of the background mean radiance (***μ***) and the negative methane absorption coefficient (***k***). To determine ***k***, we employ a forward model^62^ and convolve the radiance with the imager’s central wavelength and Full Width at Half Maximum (FWHM).^57^ The atmosphere is divided into vertical layers with a thickness of 1 km up to an altitude of 25 km, 2.5 km between 25 and 50 km, and 5 km above 50 km altitude. For the forward model simulation, methane enhancements are introduced into the lowest layer at various values, ranging from 0 to 6400 ppb in double increments of 100. The *k* value (ppb^–1^) for each band is calculated as the regression slope between the natural logarithm of the radiance and the methane enhancements. The maximum likelihood estimate of the scale factor ΔXCH_4_ is

2 $$\Delta {XCH}_{4}=\frac{{\left(t-\mu \right)}^{T}\Sigma^{-1}\left(L-\mu \right)}{{\left(t-\mu \right)}^{T}\Sigma^{-1}\left(t-\mu \right)}$$

The strong absorption window (2100–2450 nm) is selected for the ΔXCH_4_ calculation. However, the results are often noisy in urban areas (due to complicated reflectance related to, for example, roads and roofs), making it challenging to differentiate plumes from the background. To mitigate this, we perform the same retrieval over the 1300–2500 nm window,^61^ including both the strong (∼2300 nm) and weak (∼1700 nm) methane absorption windows. Then, we apply a Chambolle total variance denoising (TV) filter^63^ to obtain a smoothed ΔXCH_4_ field. The TV filter aims to minimize the cost difference between the original and smoothed images. We generate 300 plume-free noisy ΔXCH_4_ images and determine the inflection point of the threshold versus denoising weight to exclude all falsely detected plumes.^50^ Considering the lower SNR of PRISMA, we select a denoising weight of 150, higher than the weight of 50 used for EMIT and EnMAP. The two-step denoised ΔXCH_4_ field is only used for generating plume masks (Supporting Information Section S3), while the emission rate calculation employs the ΔXCH_4_ data without denoising.

### Emission Rate Quantification

Supporting Information Section S3 describes the process for generating a plume mask using the watershed technique (Figure S4).^64,65^ To account for the possibility of strong and long plumes breaking the sparsity assumption of the matched filter, we exclude the plume pixels in each column of observations. Subsequently, we rerun the retrieval process to obtain the final emission rate products. This two-step approach helps mitigate the impact of dense plumes on the background radiance estimation and typically yields higher methane emission rates.

We then apply the IME method assuming concentrated sources^66,67^ to quantify the methane emission rates (*Q* in kg h^–1^)­

3 $$Q=\frac{U_{eff}\cdot IME}{L}$$

where IME is the total methane mass (kg) in the plume mask, *L* (m) is the square root of the plume area, and *U*_eff_ is the effective wind speed (m/s). We perform instrument-specific calibrations for *U*_eff_ based on large-eddy simulations that model emissions from the landfill as an area source (Supporting Information Section S3), *U*_eff_ depends linearly on the 10 m wind speed (*U*_10_)­

4 $$EMIT:U_{eff}=0.45\cdot U_{10}+0.67$$

5 $$EnMAP:U_{eff}=0.37\cdot U_{10}+0.69$$

6 $$PRISMA:U_{eff}=0.37\cdot U_{10}+0.70$$

Our primary choice for the wind is the European Centre for Medium-Range Weather Forecasts Reanalysis 5 (ERA5) 10 m wind speed. However, we use the Goddard Earth Observing System Forward Processing (GEOS-FP) data in cases where the ERA5 wind direction differs from the plume direction by more than 90 deg. If both the ERA5 and GEOS-FP wind data fail to accurately capture the wind direction, then we default to using the ERA5 wind data.

### Climate TRACE Bottom-Up Inventory

Climate TRACE is a global greenhouse gas emissions database.^68^ The waste sector component uses Bayesian regression modeling that integrates detailed facility-level waste data from sources such as the US Environmental Protection Agency (EPA),^69^ Waste Atlas (http://www.atlas.d-waste.com/), and Global Plastic Watch (GPW; https://www.globalplasticwatch.org/), to estimate methane emissions from solid waste disposal sites globally. Observation data from remote sensing platforms, such as Carbon Mapper’s AVIRIS-aircraft observations from 2016 to 2023, are used for training purposes in developing emission data sets. The EPA data come from 2021, while the Waste Atlas data correspond to 2013, and the GPW data are obtained from 2021. Country-level emissions are generally based on EDGAR estimates, except when the sum of facility-level emissions surpasses the EDGAR-reported figure. The Climate TRACE data used in this study are from 2022 and were accessed in May 2024. All landfills detected by HSIs are classified as active in the data set.

### WasteMAP Platform

WasteMAP (https://wastemap.earth/) is an online platform that compiles waste methane emission reports, model results, and observations. We only use the city-level data estimated with the bottom-up Solid Waste Emissions Estimation Tool (SWEET) developed by the EPA. SWEET employs environmental factors and waste information from the World Bank What a Waste 2.0 report^3^ to estimate methane emissions.

## Supplementary Material





## Data Availability

The Level 1B data products for EMIT (version 1), EnMAP (version 1.4), and PRISMA (version 1) are available at the following links: https://search.earthdata.nasa.gov/search?q=C2408009906-LPCLOUD, https://www.enmap.org/data_access/, and https://prisma.asi.it/. Retrieval and emission data are available on Zenodo (10.5281/zenodo.13643544). Notebooks to reproduce this work are also deposited on Zenodo (10.5281/zenodo.14980236). HyperGas (version 0.1.0), the retrieval package, will become open-access following its publication.

## Notes

Emission uncertainty quantification; comparison with controlled releases; IME calibration and plume mask; comparison with Carbon Mapper EMIT quantifications; and detection limit (PDF)
The complete Sentinel-2 RGB time series for 2023 shown in Figure 4 (ZIP)
