# Supplementary material for: Global Identification of Solid Waste Methane Super Emitters Using Hyperspectral Satellites
Source: Environ Sci Technol. 2025 Aug 19;59(34):18134–45. doi: 10.1021/acs.est.4c14196 (PMC12409875; doi:10.1021/acs.est.4c14196)
Supplement: Supplementary file 1 [file es4c14196_si_001.pdf]

Supporting Information for "Global Identification of Solid Waste Methane Super Emitters using Hyperspectral Satellites"

Xin Zhang,[*,†] Joannes D. Maasakkers,[†] Javier Roger,[‡] Luis Guanter,[‡,¶] Shubham Sharma,[†] Srijana Lama,[†] Paul Tol,[†] Daniel J. Varon,[§] Daniel H. Cusworth,[‖] Katherine Howell,[‖] Andrew K. Thorpe,[⊥] Philip G. Brodrick,[⊥] and Ilse Aben[†,#]

†*SRON Space Research Organisation Netherlands, Leiden, 2333 CA, The Netherlands*

‡*Research Institute of Water and Environmental Engineering, Universitat Politècnica de València, Valencia 46022, Spain*

¶*Environmental Defense Fund, Amsterdam, 1083 HN, The Netherlands*

§*School of Engineering and Applied Sciences, Harvard University, Cambridge, 02138, MA, USA*

‖*Carbon Mapper, Pasadena, 91105, CA, USA*

⊥*Jet Propulsion Laboratory, California Institute of Technology, Pasadena, 91109, CA, USA*

#*Department of Earth Sciences, Vrije Universiteit Amsterdam, Amsterdam, 1081 HV, The Netherlands*

E-mail: xin.zhang@sron.nl

Contents

S1 Emission Uncertainty Quantification **S3**

S2 Comparison with Controlled Releases **S5**

S3 IME Calibration and Plume Mask **S5**

S4 Comparison with Carbon Mapper EMIT Quantifications **S10**

S5 Detection Limit **S11**

S6 Supplementary Figures and Tables **S12**

References **S30**

Number of figures: 18

Number of tables: 8

S1 Emission Uncertainty Quantification

There are three sources of uncertainty in our emission uncertainty estimations: wind speed error, retrieval random error, and uncertainty in the integrated mass enhancement (IME) calibration.[1–3] For the error in the wind speed, we compare the European Centre for Medium-Range Weather Forecasts Reanalysis 5 (ERA5) 10-m wind data with the Automated Surface Observing System (ASOS) dataset obtained from worldwide airports (`https://mesonet.agron.iastate.edu/ASOS/`). We only include the wind data recorded between 10:00 and 14:00 (local time) to coincide with HSI overpass times. The standard deviation of the difference between ERA5 and ASOS wind data, is ~1.5 m s^{-1} for wind speeds higher than 3 m/s. For wind speeds lower than 3 m/s, we apply a relative wind error of 50%.[4] We also compare the ERA5 and GEOS Forward Processing (GEOS-FP) wind reanalysis data and find that their difference falls within our wind uncertainty estimate.

To quantify the effects of retrieval random error, we apply the plume mask to non-plume pixels across the entire scene and calculate the standard deviation of the emission rates.[1] The last component of uncertainty is the IME calibration (Section S3) error. The area-source calibration that we use assumes a uniform distribution of methane emissions across a 275 × 275 m^2 area, whereas the real distribution can be more complex.[3] To estimate the uncertainty originating from this simplification, we change the effective wind calibration to one that is calibrated using point sources and calculate the resulting change in emission rate.[3]

Overall, the uncertainties associated with wind speed error, retrieval random error, and IME calibration error are 24%, 15%, and 16%, respectively (Fig. S1). To estimate the uncertainty in individual estimates or summation of methane emissions from different landfills, we calculate the square root of the sum of the squares of the individual uncertainties.

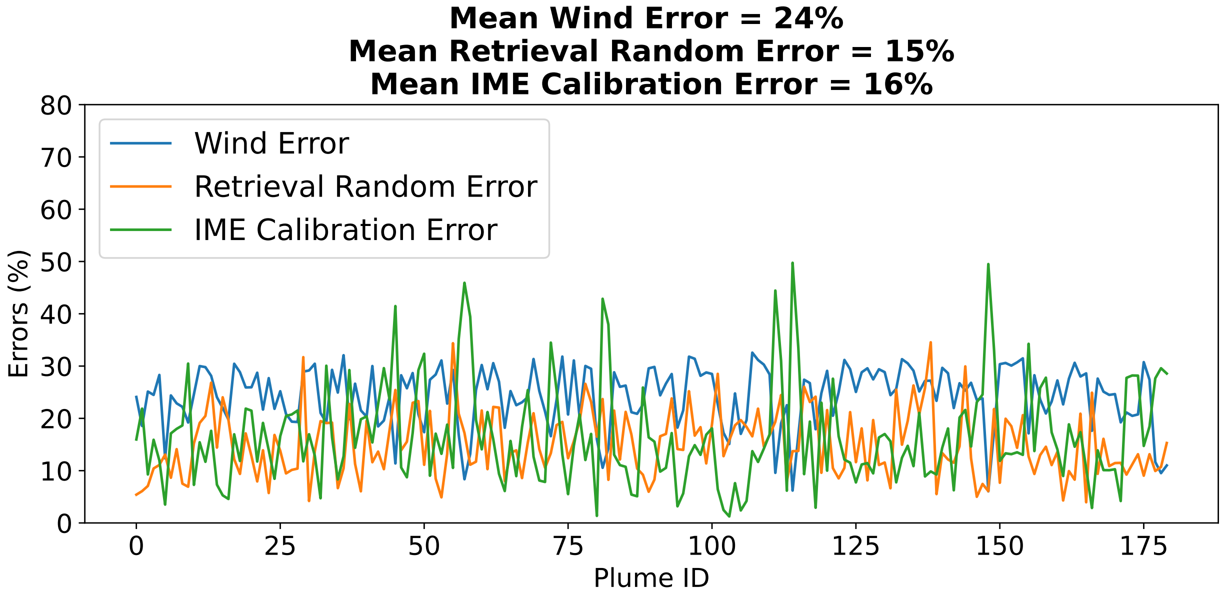

Figure S1: Relative estimation uncertainties from wind (blue), retrieval random error (orange), and IME calibration error (green). The wind error is set as 1.5 m s^{-1} for wind speeds higher than 3 m/s, while it is 50% for wind speeds lower than 3 m/s. The random error is estimated using the standard deviation of emission rates obtained by shifting the plume mask to non-plume pixels across the entire scene. The plume IDs on the x-axis are arranged chronologically.

S2 Comparison with Controlled Releases

We validate our emission quantification by comparing the derived emission rates based on ERA5 wind speed data with controlled methane releases conducted in 2021 and 2022 (Fig. S2). For the EnMAP controlled release, the actual release rate was 1.1 t h^{-1}, while our estimation yields 1.6 ± 0.5 t h^{-1}, which agrees with the estimations from other analysis teams ranging from 1.5 to 1.8 t h^{-1}.[5] Similarly, for the PRISMA controlled release, our estimation is 5.2 ± 1.8 t h^{-1}, while the actual release rate was 4.5 t h^{-1}, and other analysis teams estimated emission rates within the range of 3.6 to 5.0 t h^{-1}.[6]

Although on-site wind measurements are available for these two controlled releases, instantaneous data may not accurately represent methane plume turbulence, particularly for elongated plumes (e.g., the 2021 release). Figure S2 C–D shows that the ERA5 wind speed falls within the 2–8 m/s of observed wind speeds near the overpass times. Our estimates, including wind uncertainty, are consistent with the release rates (Section S1). Future area-source validations will require careful consideration of ground wind profile measurements to accurately derive the wind transporting the plume.

S3 IME Calibration and Plume Mask

To calibrate the effective wind speed used in the IME calculation against reanalysis 10 m wind speeds, we employ Weather and Research Model large-eddy simulations (WRF-LES) for two source types: a 275 × 275 m^2 area source (e.g., like a landfill[3]) and a point source (e.g., oil & gas and underground coal mining facilities). We randomly scale source rates from 1 to 30 t h^{-1} and add normally distributed measurement noise (Fig. S3A). Noise levels are defined by standard deviations of non-plume methane enhancement in clear-sky hyperspectral scenes, with precisions of 3%, 5%, and 12% for EMIT, EnMAP, and PRISMA, respectively. For each plume, the effective wind speed (U_{eff}) is computed from QL/IME, where the emission rate (Q) is known, and plume length (L, square root of the plume area)

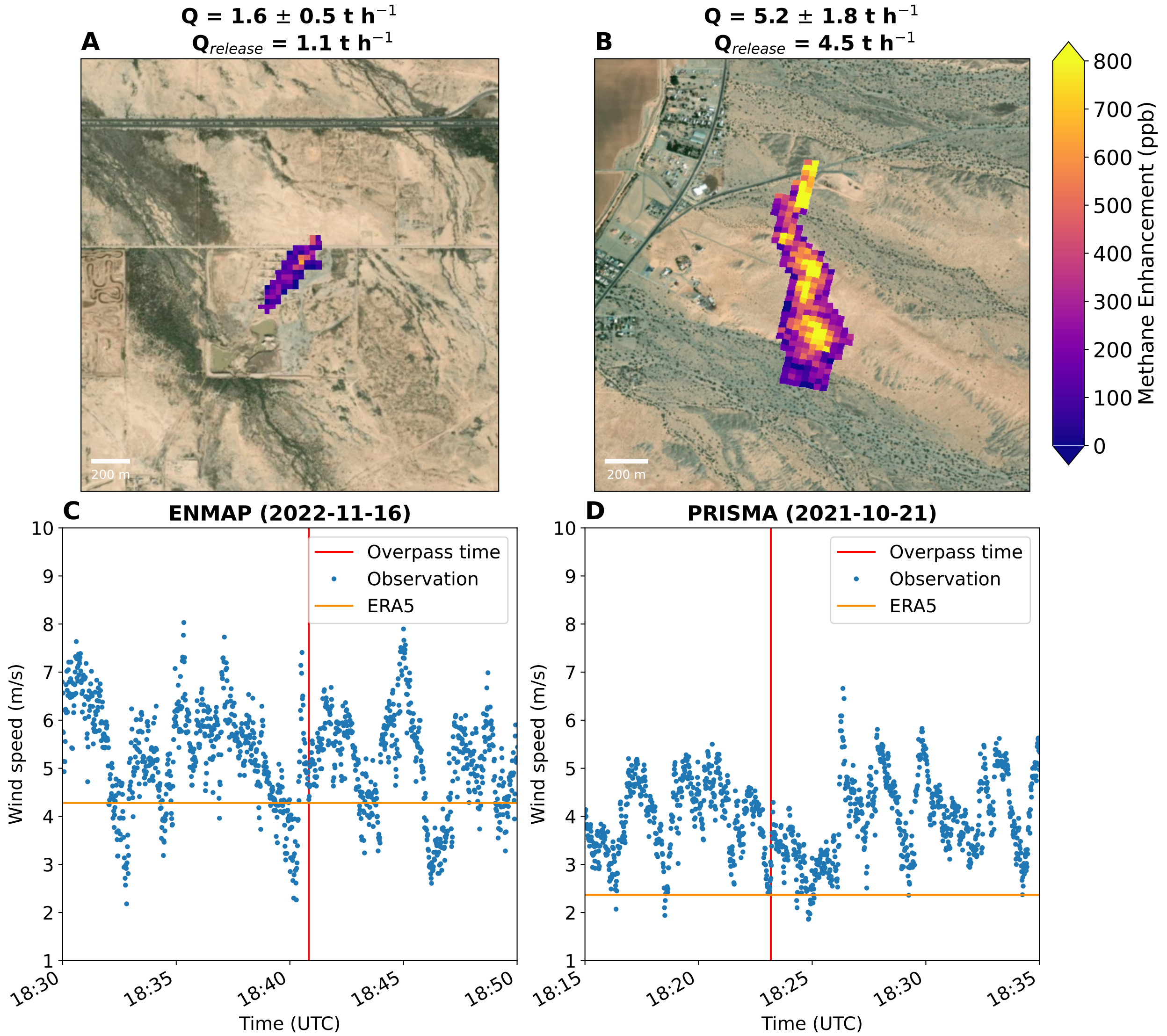

Figure S2: Methane enhancements observed by (A) EnMAP on November 16, 2022, and (B) PRISMA on October 21, 2021, for two controlled methane release experiments.[5,6] Our estimates 1.6 ± 0.5 t h^{-1} and 5.2 ± 1.8 t h^{-1} compare well with the actual releases of 1.1 t h^{-1} and 4.5 t h^{-1} respectively. The release sites are marked with a white 'x'. Background imagery reproduced with permission from Esri World Imagery.[7] (C–D) Wind speed comparisons between measured data[5,6] and ERA5 reanalysis. Red lines are satellite overpass times, and orange lines show ERA5 wind speeds.

and IME are calculated from plume masks.

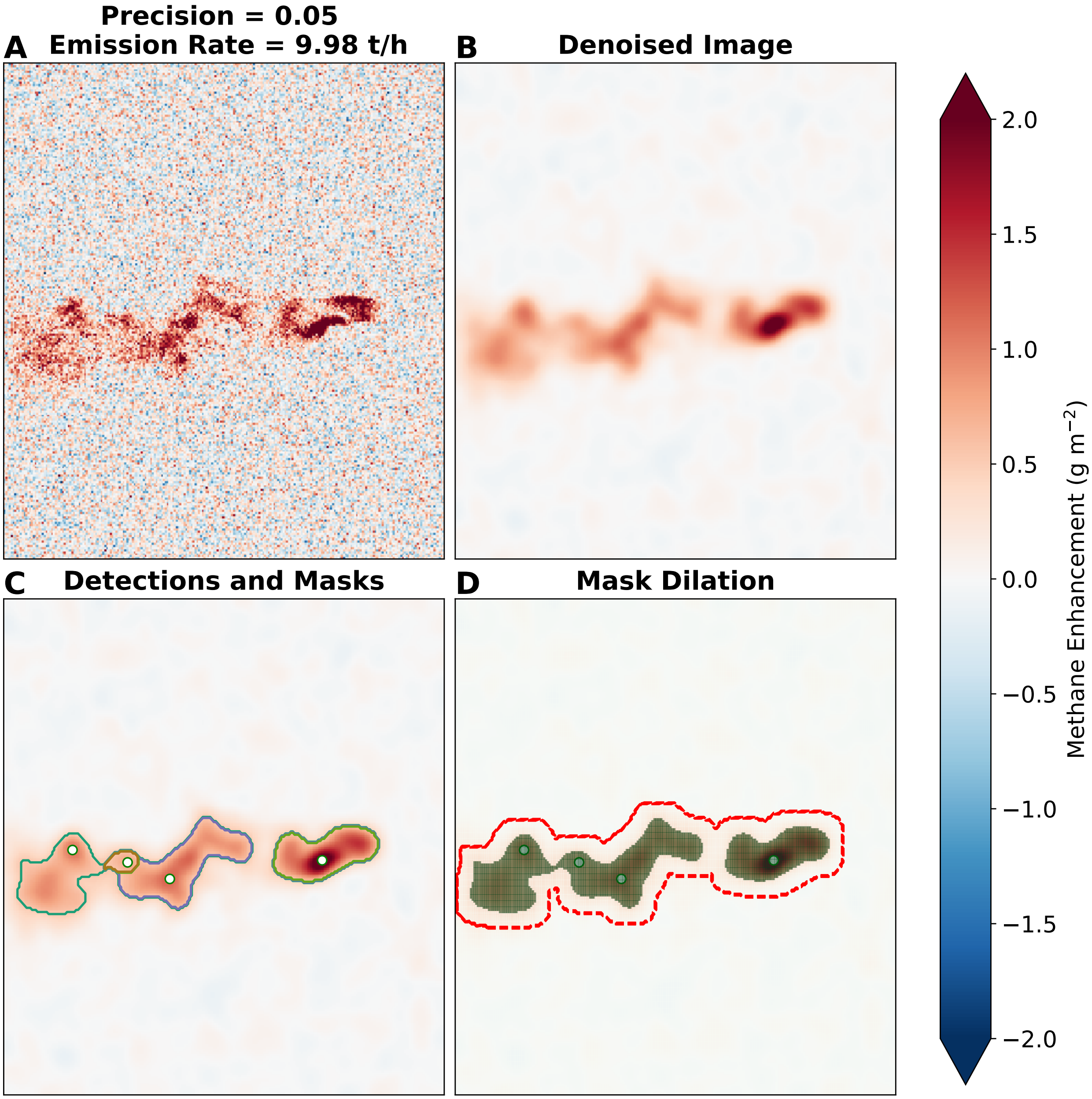

Figure S3: Plume mask generation process for methane emissions using WRF-LES simulation. (A) Methane enhancement (ΔXCH_4) with added Gaussian noise (σ=0.05×1875 ppb). (B) Denoised ΔXCH_4 field after applying a Chambolle total variation (TV) denoising filter. (C) Initial plume masks derived from the watershedding algorithm. White dots indicate high-ΔXCH_4 locations; contours represent individual masks. (D) Final plume mask (dark green): initial masks expanded by 180 m and combined (red).

We derive methane plume masks by applying a watershedding technique to denoised methane fields (Fig. S3B). This method has been applied to track convective clouds[8] and

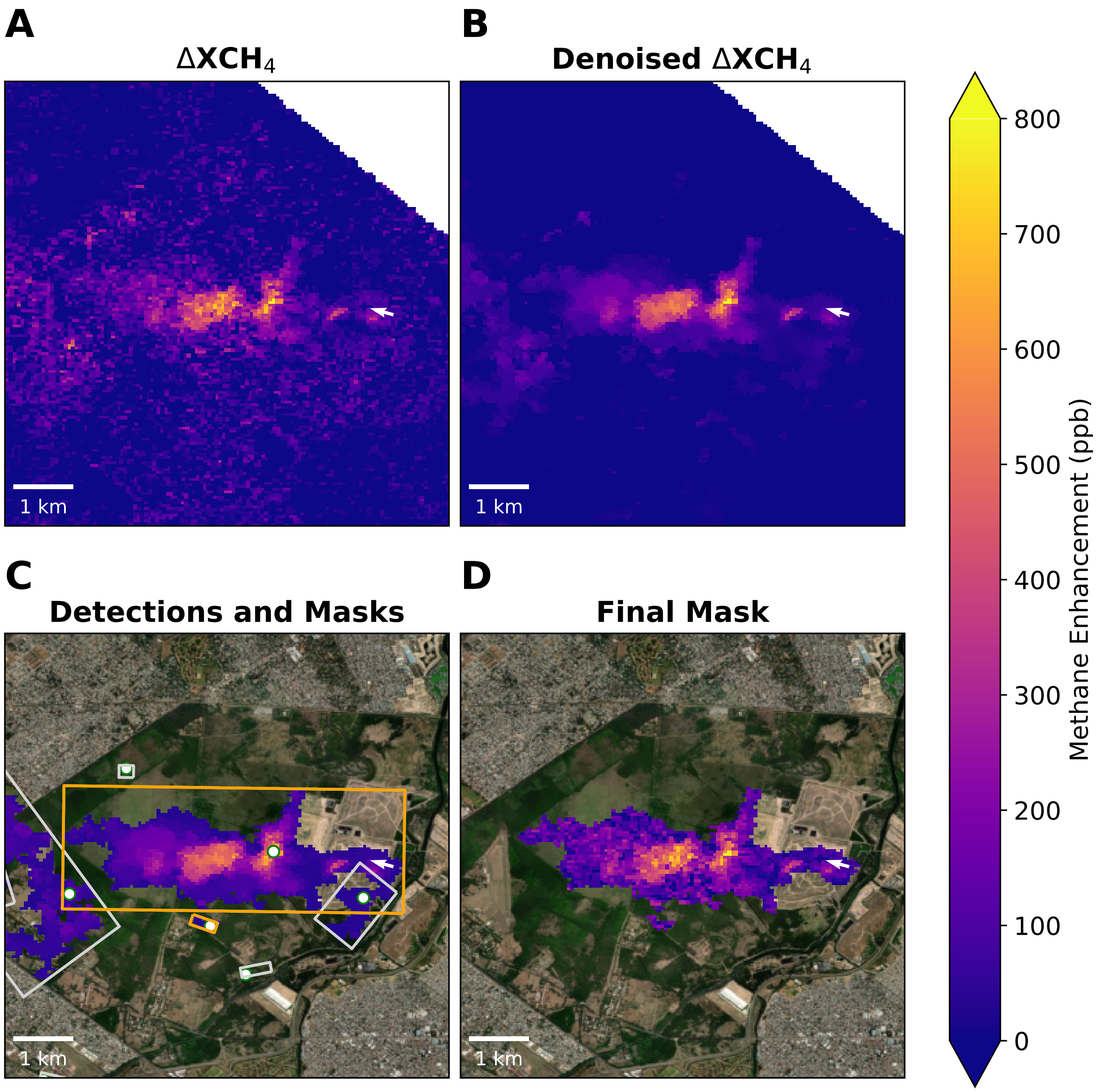

Figure S4: Plume mask creation process for the Norte III landfill methane emission using the EMIT observation on November 24, 2023. The white pixels represent missing data (outside the EMIT image swath), while the white arrow indicates the ERA5 wind direction. (A) Methane enhancement (ΔXCH_4) derived from the strong CH_4 absorption window (2100∼2450 nm). (B) Denoised ΔXCH_4 field obtained by applying the Chambolle total variance denoising (TV) filter to ΔXCH_4 within the 1300∼2500 nm window. (C) Initial plume masks derived from watershedding algorithm. White dots indicate high-ΔXCH_4 locations; rectangles represent the minimum rotated rectangles for each mask, with orange rectangles indicating azimuth differences less than 30°. (D) Final ΔXCH_4 plume mask.

nitrogen dioxide plumes in TROPOMI observations.[9] It treats pixel values as a topographic surface and separates them into catchment basins. Threshold values of 2 and 3 standard deviations are used to identify multiple localized high-enhancement features and nearby areas with high enhancement values (Fig. S3C). We dilate these masks by 180 m and merge overlapping masks, with the mask containing the emission source used to identify masks from a single source (Fig. S3D). Figure S4 demonstrates the plume mask determined for a Norte III landfill methane emission plume. To ensure plumes originate from the same source, we limit the azimuth difference of the oriented envelope (minimum rotated rectangle) to less than 30° (Fig. S4C), assuming minimal wind direction changes around the landfill. Non-detects are classified if no plume mask covers the source of interest.

Figure S5 shows the relationship between U_{eff} and U_{10} inferred from the LES ensemble. We use the area-source calibration by default and the point-source calibration to estimate calibration error.

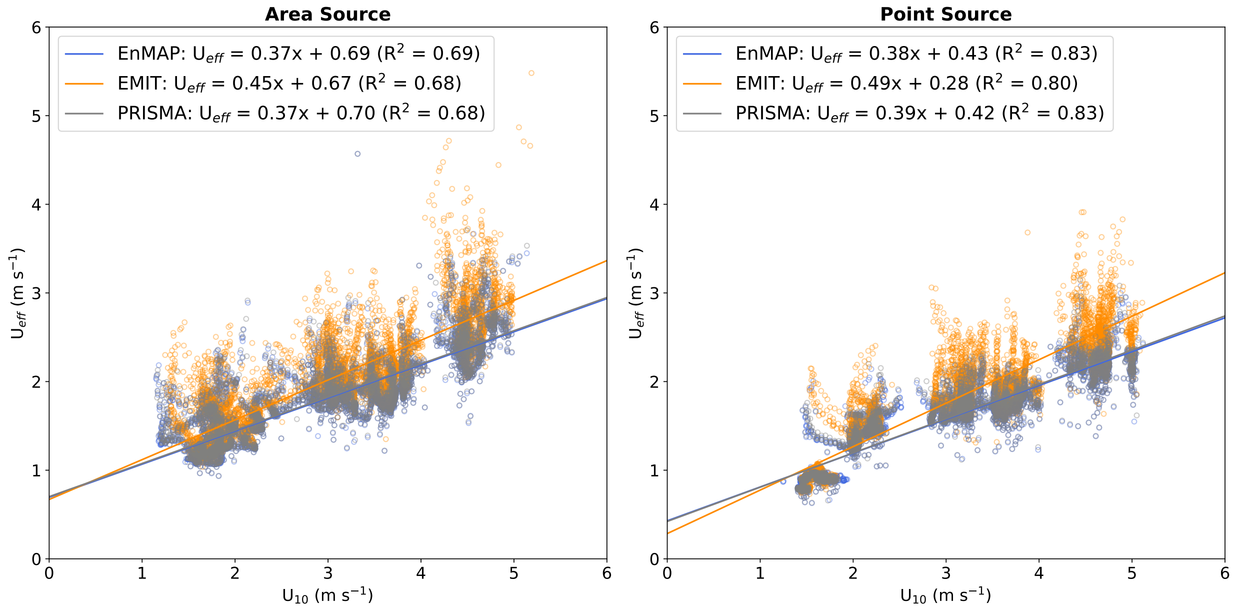

Figure S5: Relationship between the effective and local 10 m wind speeds for different instrument precisions and source types based on WRF LES simulations.

S4 Comparison with Carbon Mapper EMIT Quantifications

Carbon Mapper (https://data.carbonmapper.org) provides methane emission rate estimates for EMIT using a method we call 'IME-fetch', which only uses the first 2500 m of the plume to perform the quantification. We apply this method and compare the results to our IME results. The IME-fetch method consists of the following steps: 1) Center the Level 2B methane enhancement map on the plume origin, covering an area of ± 2500 m in both horizontal directions. 2) Use a 90th percentile threshold with a 1000 m crop to distinguish between the background and plume enhancements. Identify pixels exceeding this threshold and group them into connected clusters. Consider only clusters with at least 5 pixels as part of the plume. 3) Apply a proximity criterion to each cluster group, excluding separated clusters more than 15 pixels away from the plume origin. The emission rate is calculated as IME-fetch$\cdot U_{10}/L$, where U_{10} is the mean 10 m wind speed in the plume mask (the method does not rely on an effective wind speed) and L is the maximum distance from the plume origin to another point along the segmented plume's convex hull.

Figure S6A compares source rates retrieved from both IME and IME-fetch methods to the true source rates from WRF-LES. While the IME method shows good agreement (slope=0.99, R^2=0.93) due to calibration, the IME-fetch results underestimate the emission rates (slope=0.77, R^2=0.89). This disagreement is mainly due to differences in used plume length (Fig. S6B), which depends on the plume masking method. Our IME method (Section S3) uses a smoother plume mask without fetch distance limitations, leading to more plume pixels for longer plumes. This trend is also observed in real EMIT observations (Fig. S6C), but with greater magnitude. Further research is needed to accurately reproduce both trend and magnitude, which will help address potential biases in quantification.

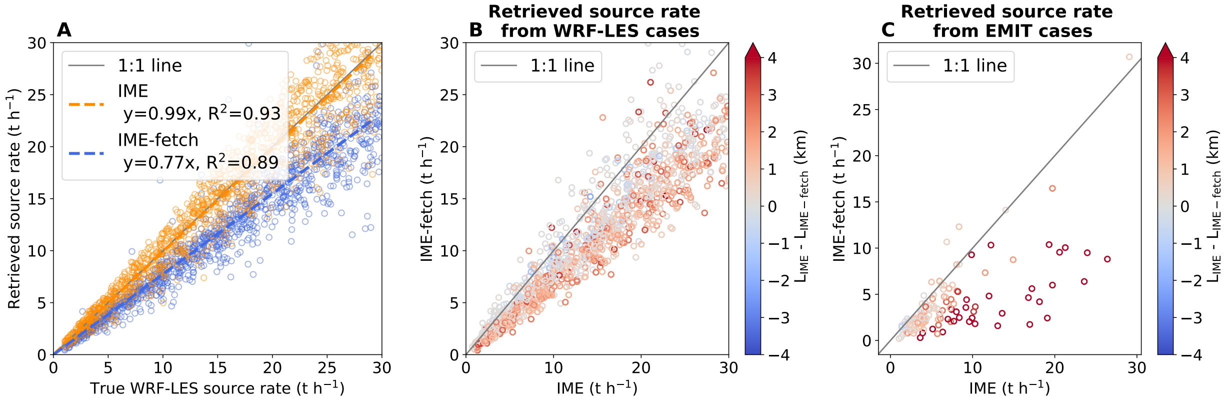

Figure S6: (A) Comparison of the IME (this study) and IME-fetch (Carbon Mapper) methods for estimating source rates using the WRF-LES test set for EMIT. (B) Correlation between IME and IME-fetch values as a function of plume length difference. (C) Same as (B), but from 127 EMIT observations over 36 landfills in this study.

S5 Detection Limit

The theoretical point-source methane detection limit (Q_{min}) of instruments can be derived from:

$$Q_{min} = PUGq \tag{1}$$

where P is the methane precision (kg m^{-2}, see Section S3), U is the mean wind speed (3 m s^{-2} used here), G is the ground sampling distance (m), and q is a constant equal to 5 for quantification.[10,11] This results in detection limits of 810 kg h^{-1} for EnMAP and 970 kg h^{-1} for EMIT. For the EnMAP observations in this study, we find one plume with an emission rate below 1 t h^{-1} and 8 plumes with emission rates between 1 and 2 t h^{-1}. The EMIT data show 10 plumes with emission rates between 1 and 2 t h^{-1}, but none below 1 t h^{-1}.

S6 Supplementary Figures and Tables

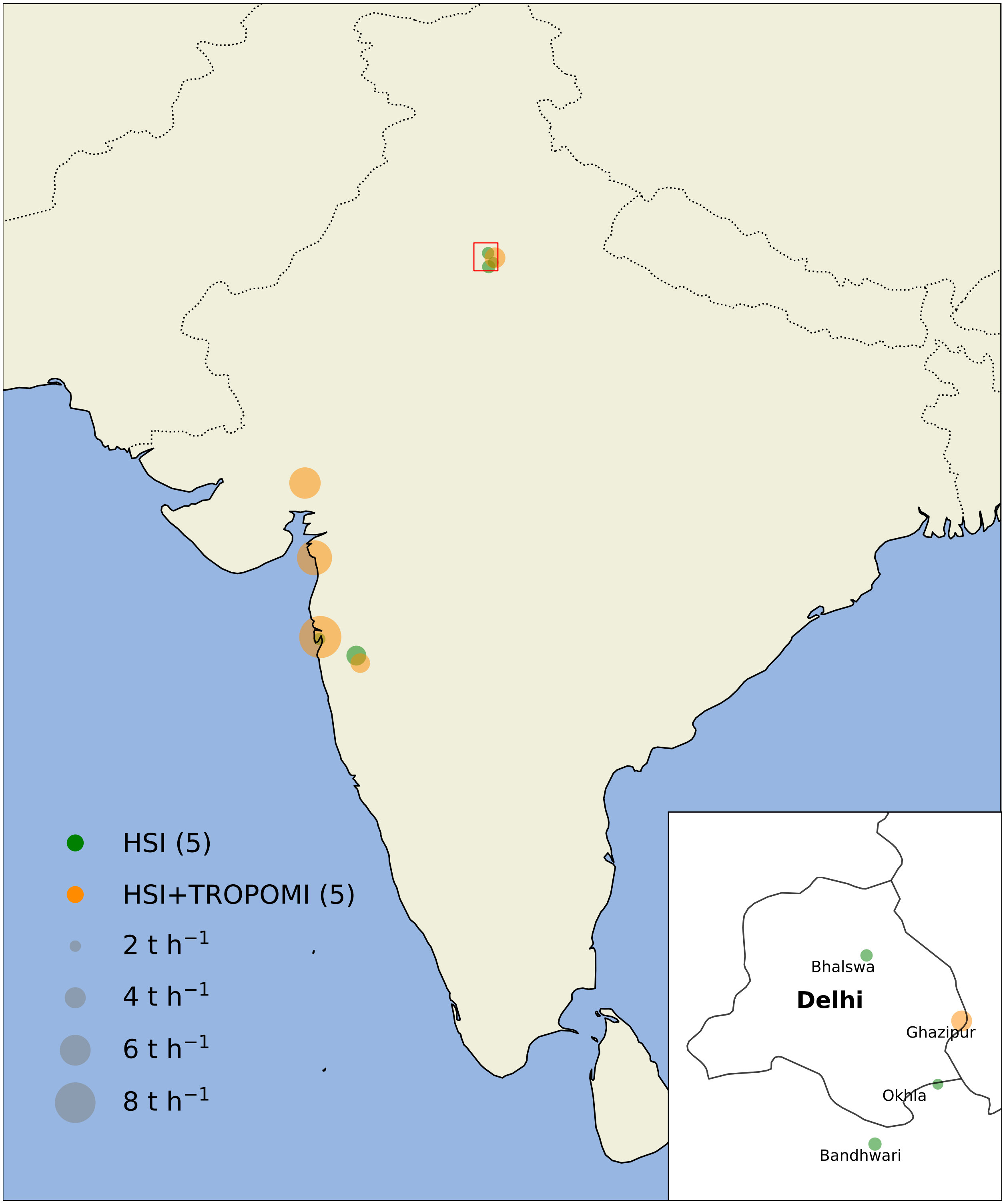

Figure S7: Landfill emissions detected by HSI across India, with a zoomed-in view of the Delhi region.

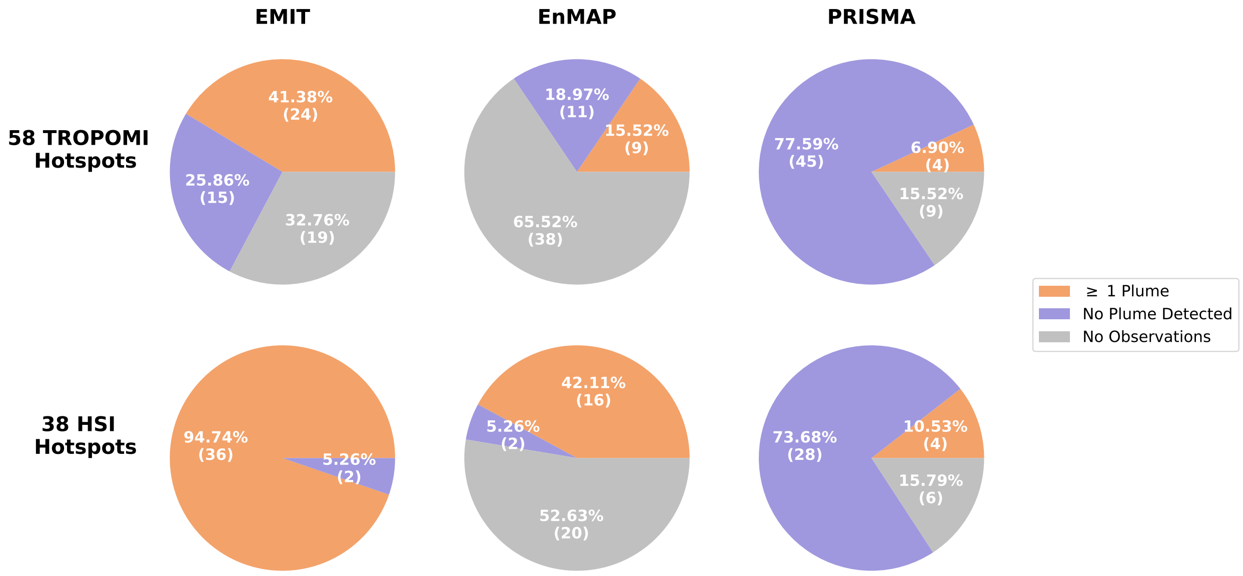

Figure S8: Variation in landfill hot spots detection efficiency by different HSIs (EMIT, EnMAP, and PRISMA) distinguishing three categories: detection of at least one plume (orange), clear-sky observations without detected plumes (purple), and no clear-sky observations (grey). Corresponding percentage values are displayed next to the number of hot spots in each category.

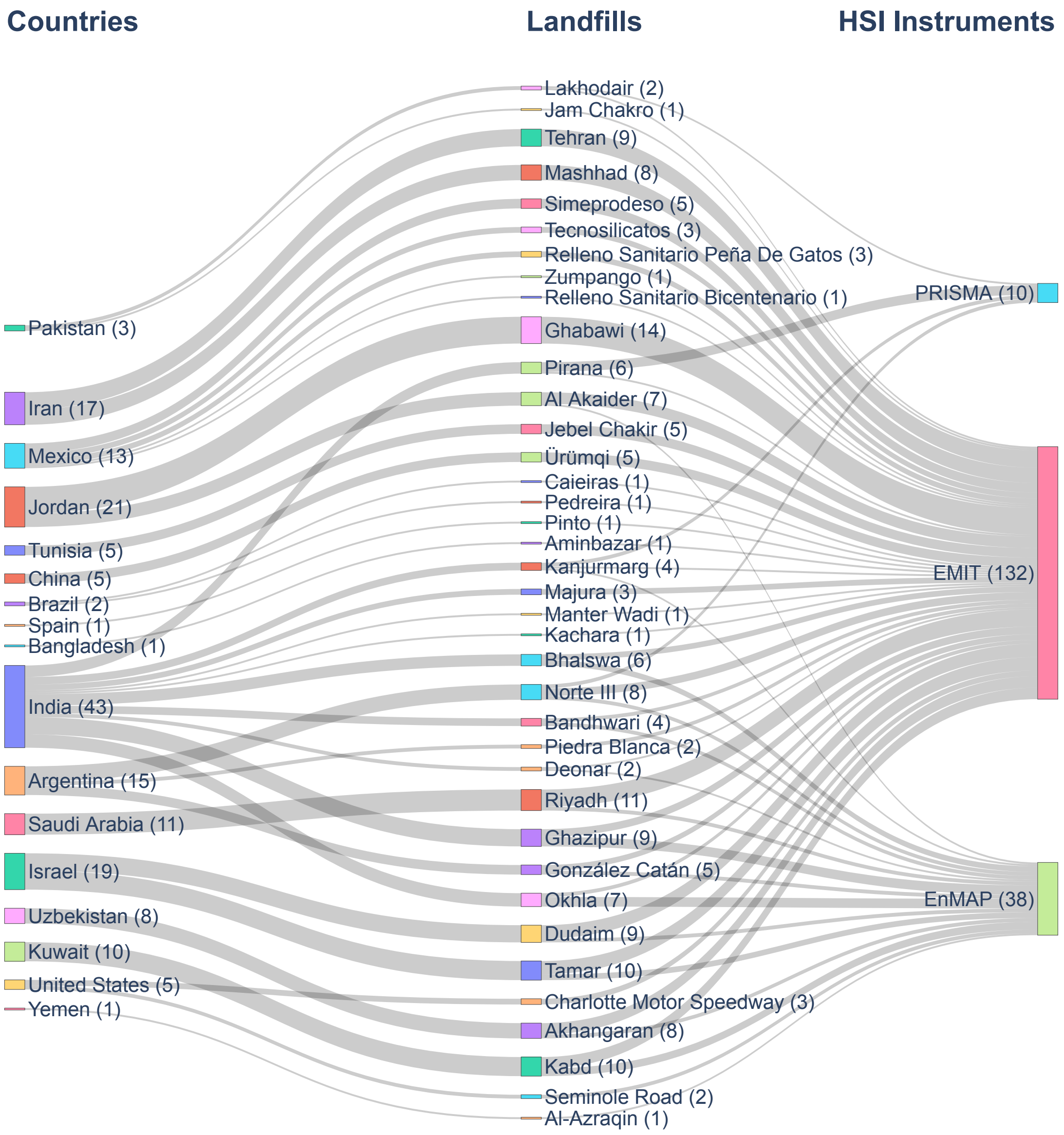

Figure S9: Sankey plot for the numbers of landfill plumes detected by HSIs (EMIT, EnMAP, and PRISMA). The numbers beside each country represent the total number of plumes detected from landfills within that country; the numbers next to each landfill indicate the number of detected plumes, and the numbers on the right show the total observations per HSI instrument.

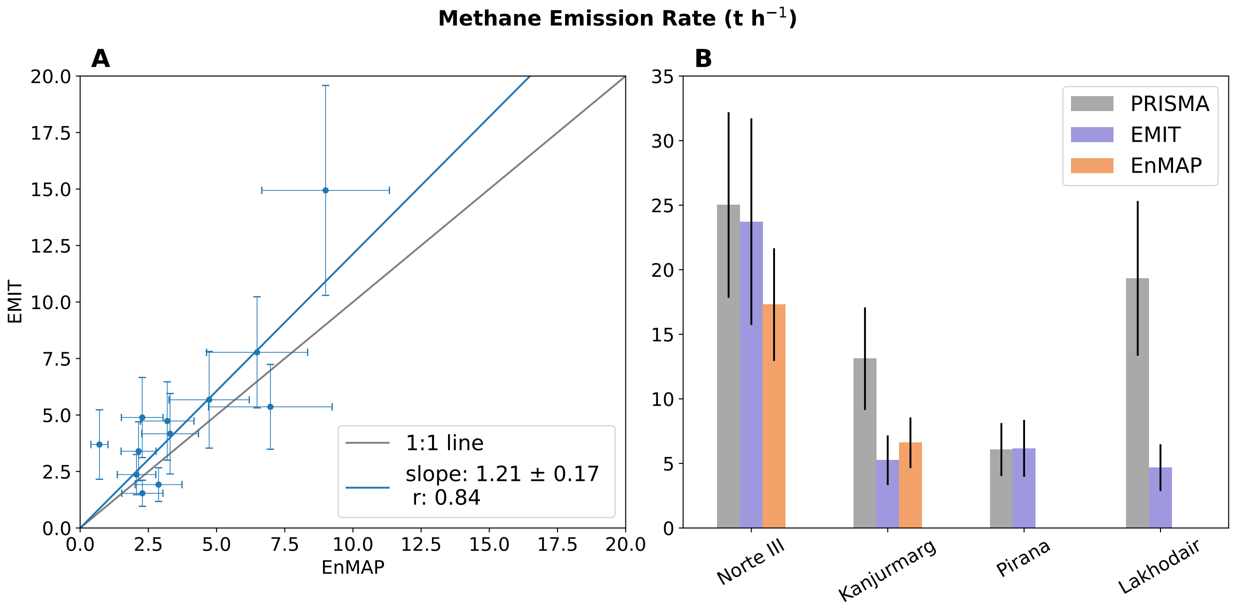

Figure S10: Comparison of average methane emission rates estimated with different HSIs for the same 24 landfill sites. (A) The orthogonal distance regression between methane emission rates estimated using the EMIT and EnMAP HSI sensors. (B) The methane emission rates of the four landfills with methane plumes detected by PRISMA. Observations were made by EMIT and EnMAP in 2023 for all sites. PRISMA observations were from 2023 for the Norte III and Pirana landfills, and 2020–2022 for the Kanjurmarg and Lakhodair landfills.

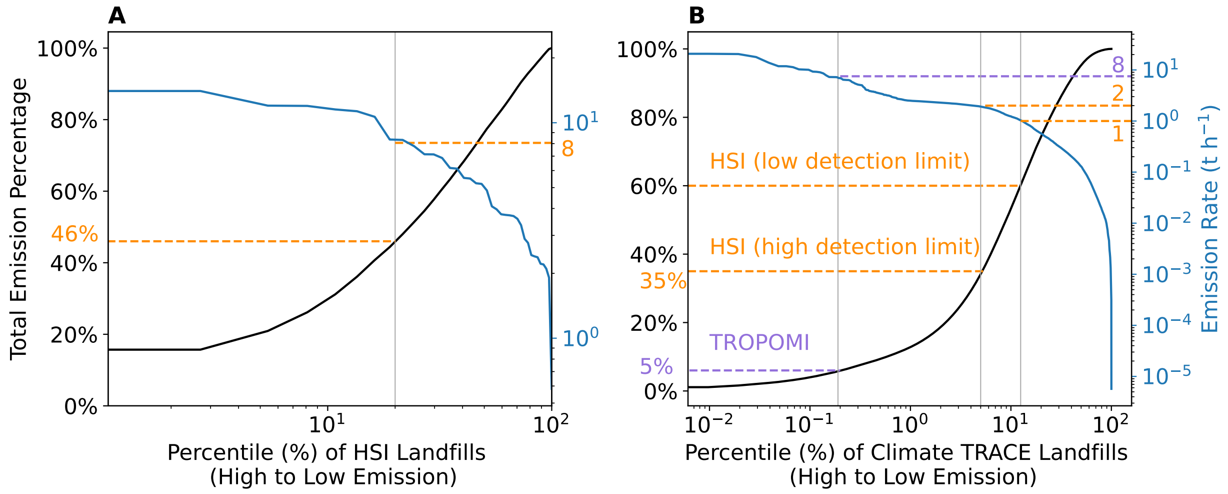

Figure S11: Cumulative distributions of landfill methane emissions. The black lines represent the cumulative distribution function of summed emission rates across landfill percentiles (in descending order), while the blue line indicates the emission rates at each respective percentile. (A) Landfills identified by HSIs. The top 20% of the highest emitting landfills emit 46% of total HIS-detected landfill emissions. (B) Landfills in the Climate TRACE dataset. The 1–2 t h^{-1} limit (orange line) and the 8 t h^{-1} limit (purple line) correspond to the estimated detection thresholds of HSI and TROPOMI, respectively.

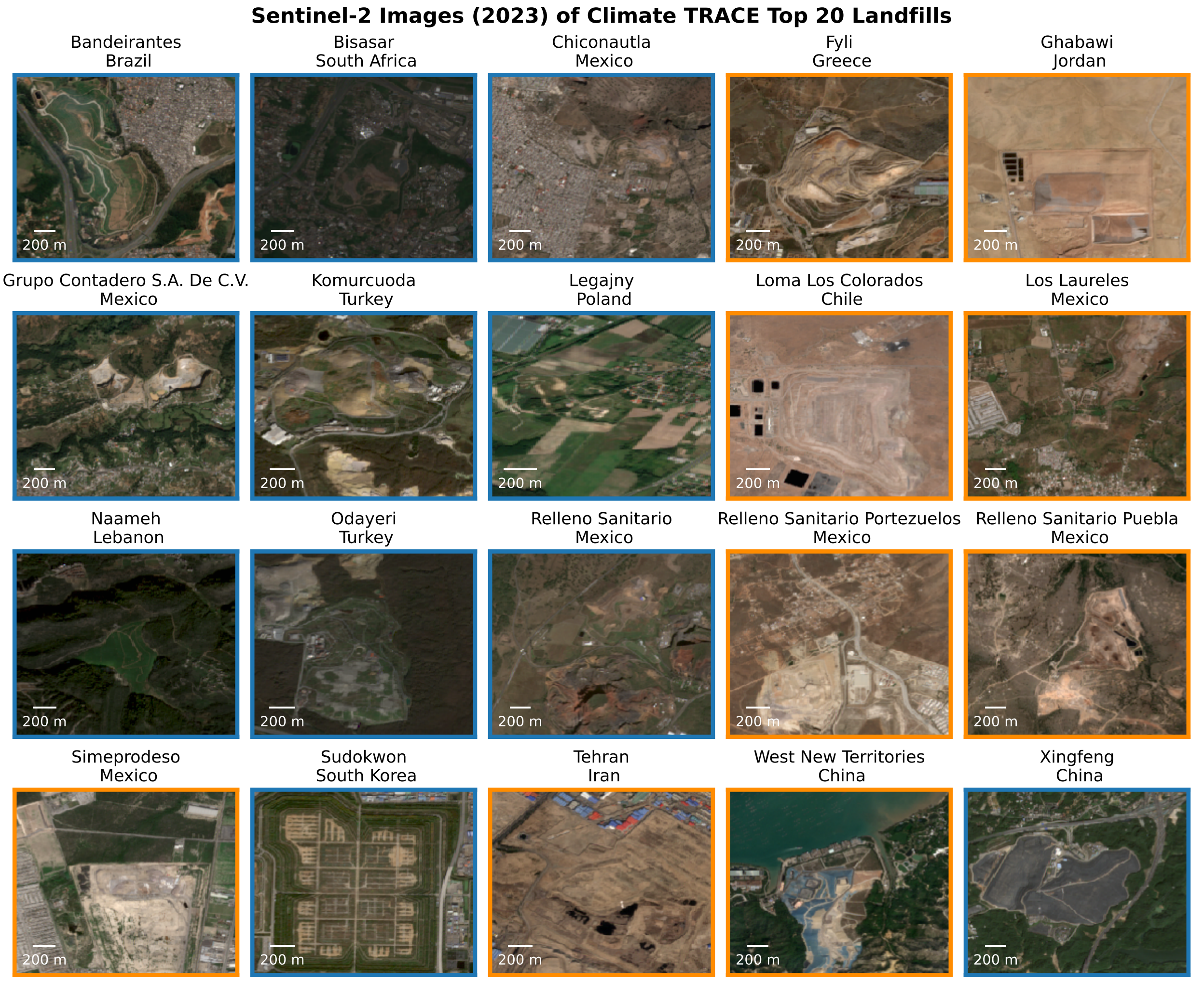

Figure S12: Sentinel-2 satellite images from 2023[12] showing the top 20 emitting landfills identified in the Climate TRACE dataset. An orange frame indicates that the HSIs detected methane plumes, while a blue frame means they did not.

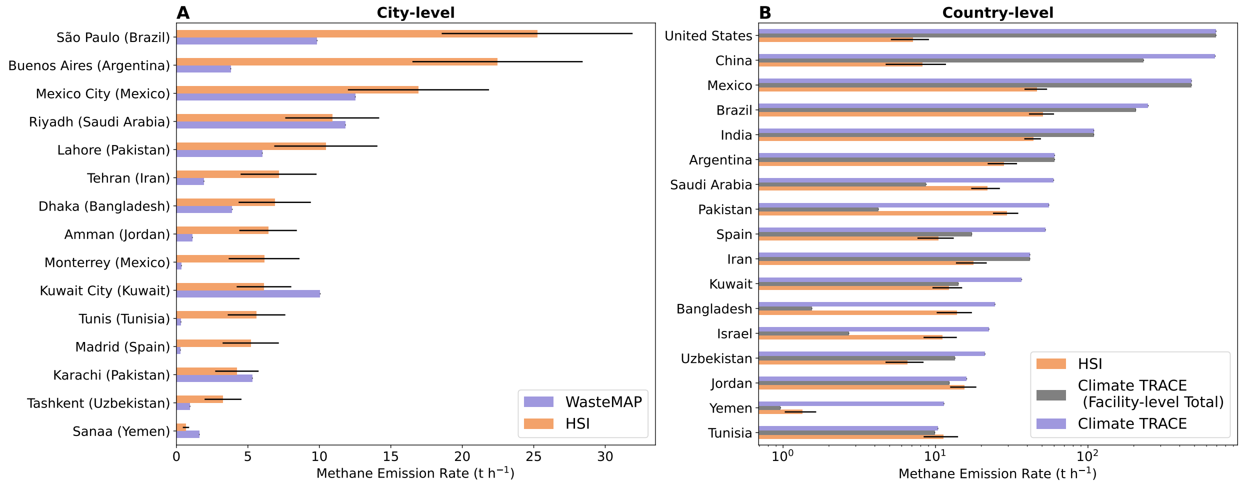

Figure S13: Comparison of methane emissions from landfills summed at the (A) city and (B) country levels, estimated using HSI observations, WasteMAP, and Climate TRACE inventories. The emission rates calculated using HSI represent the total emissions from measured and analyzed landfills in each city and country (Table S7 and S8). The total facility emissions for each country (not just the landfills analyzed using the HSI), as reported by Climate TRACE, are shown in gray.

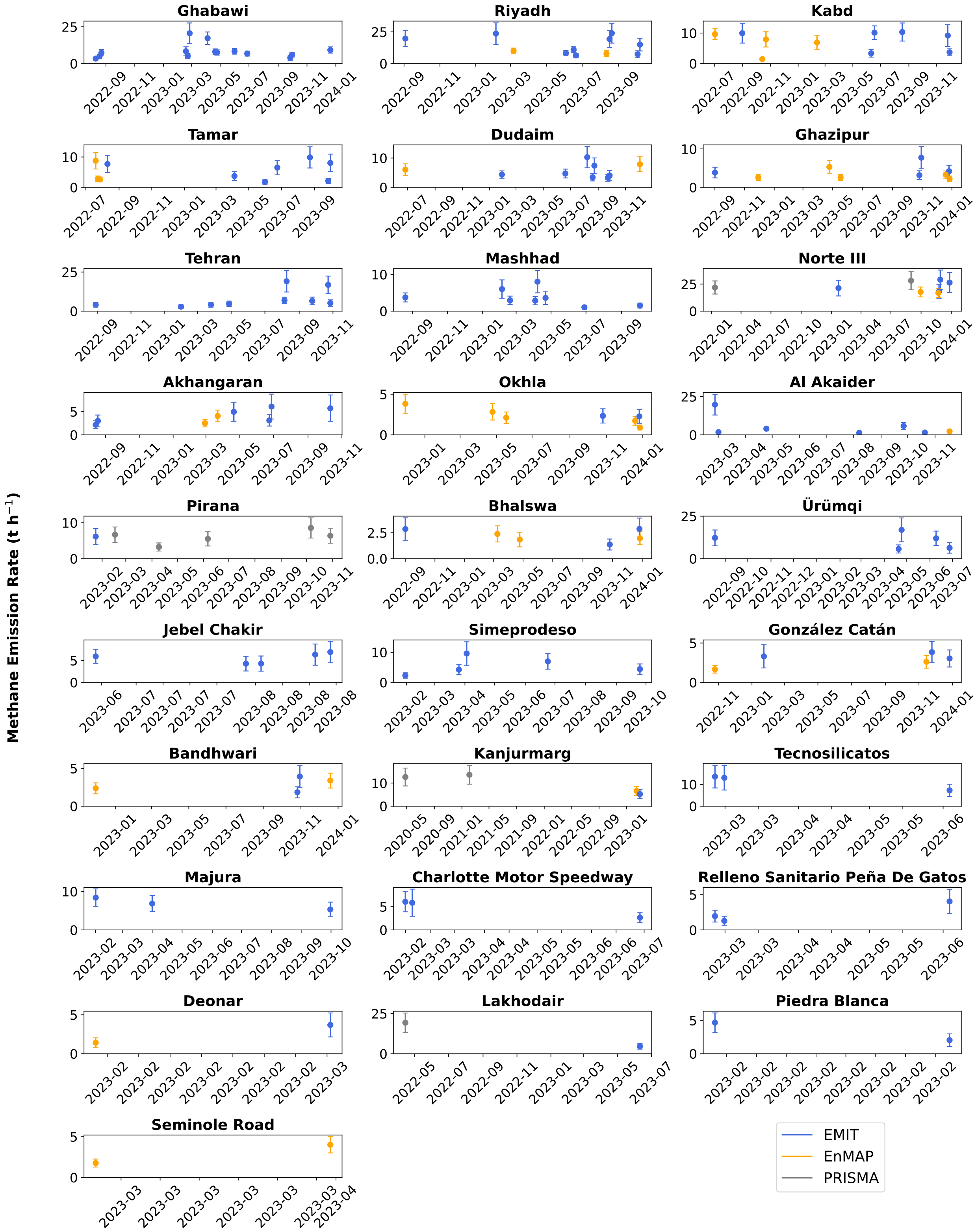

Figure S14: Time series of methane emission rates from landfills detected at least once with HSIs.

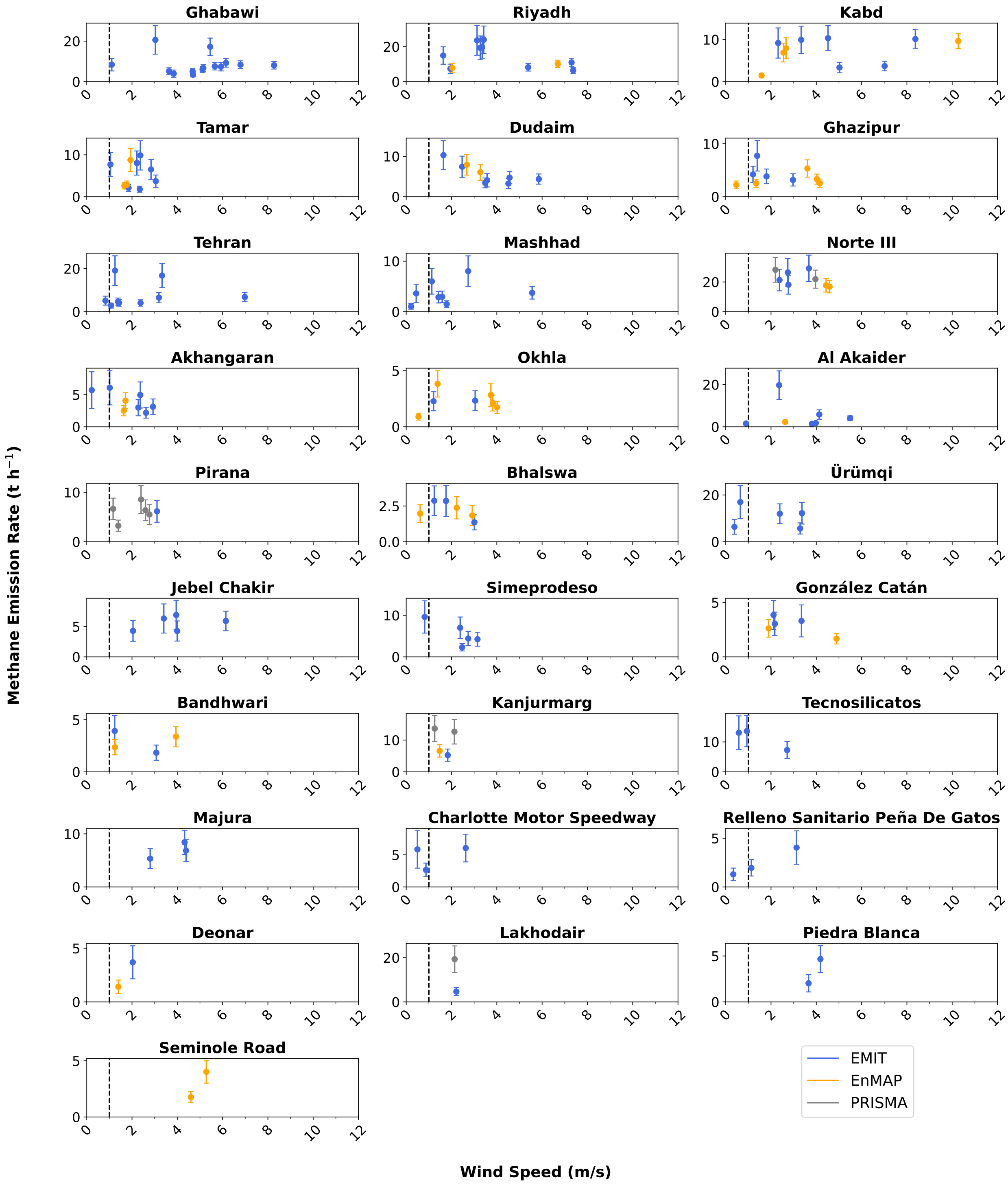

Figure S15: Relationship between wind speed and methane emission rates from landfills detected at least once with HSIs.

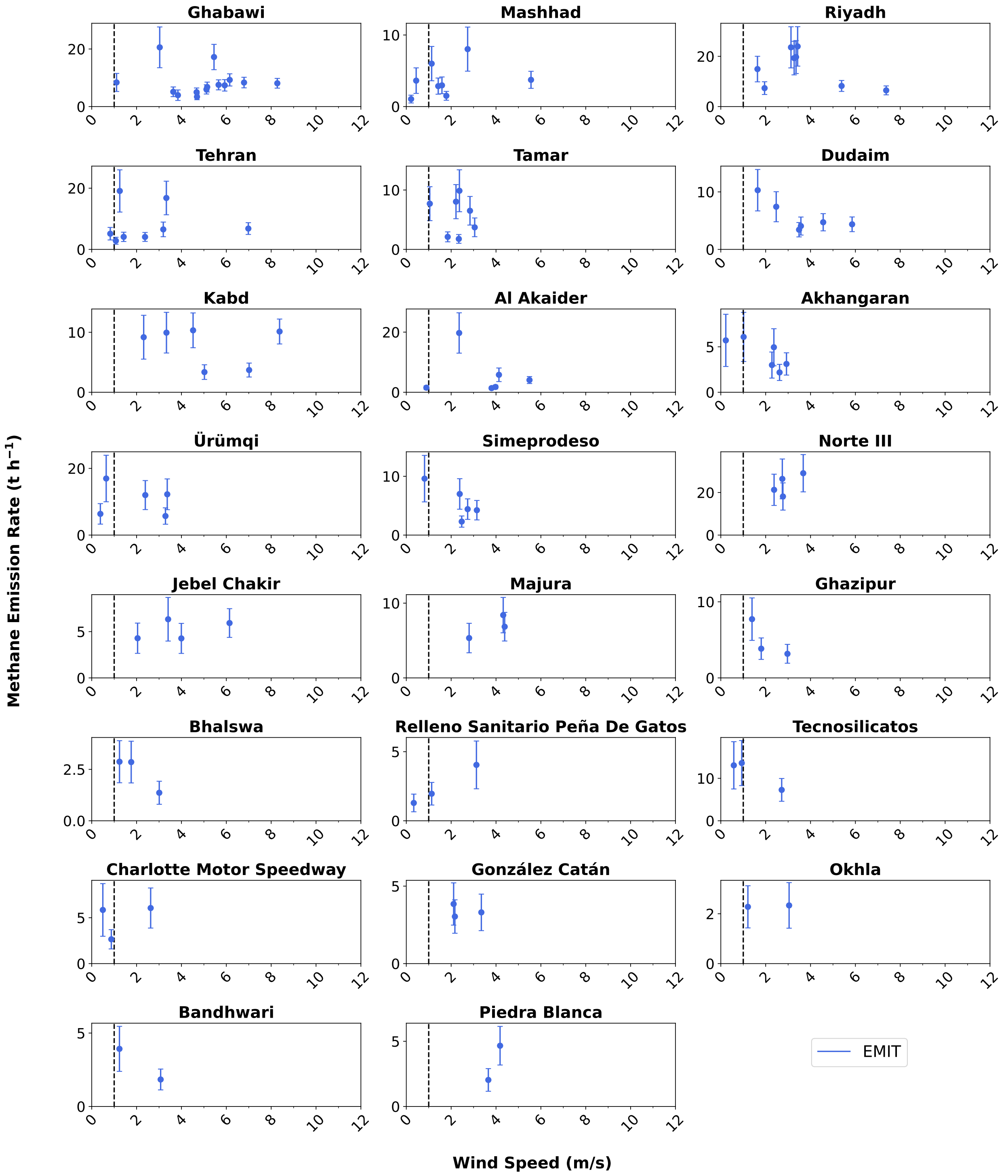

Figure S16: Same as Fig. S15, but showing emission estimates derived from EMIT data using the IME-fetch method.

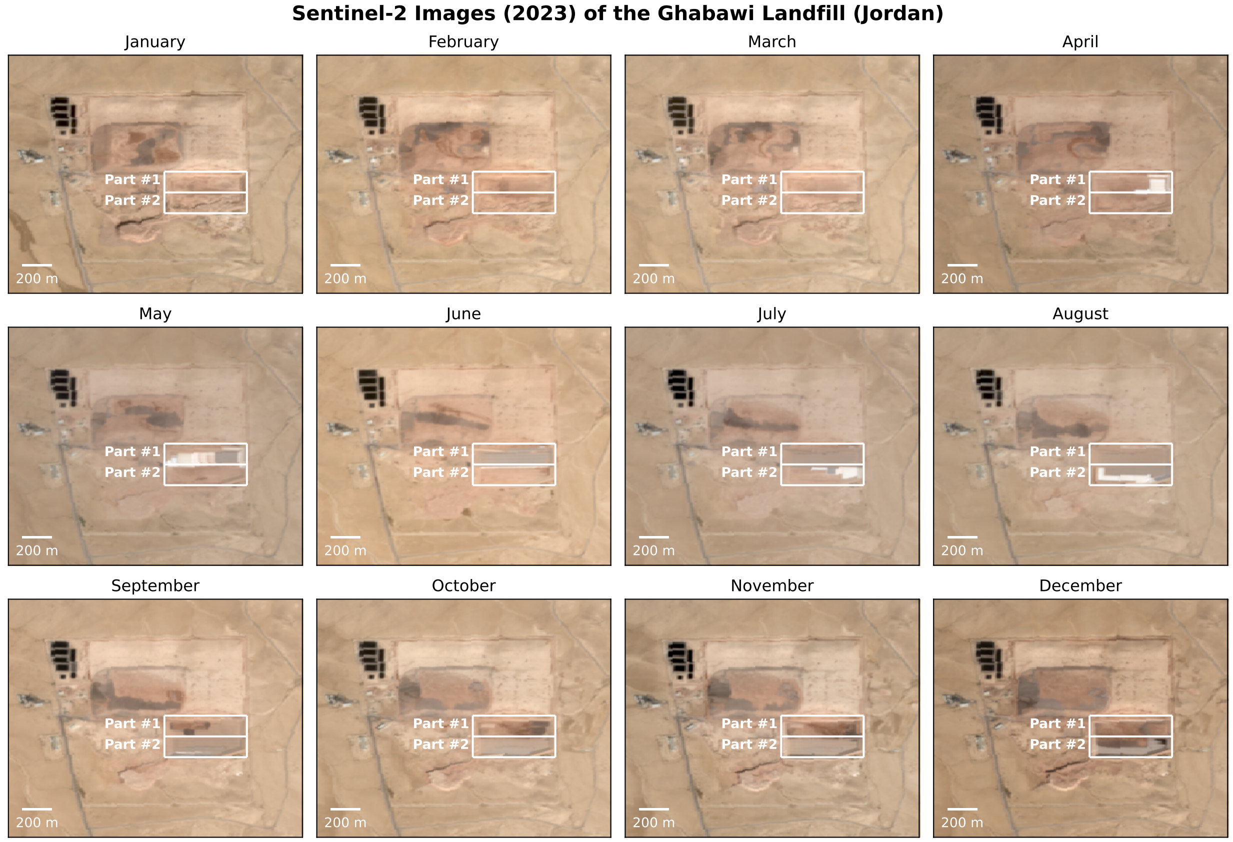

Figure S17: Monthly Sentinel-2 RGB images[12] captured in 2023 showing the Ghabawi Landfill in Jordan. The two white rectangles highlight two cells within the recently developed southern section. Movie S1 shows a time-lapse sequence of all cloud-free Sentinel-2 RGB images captured throughout 2023.

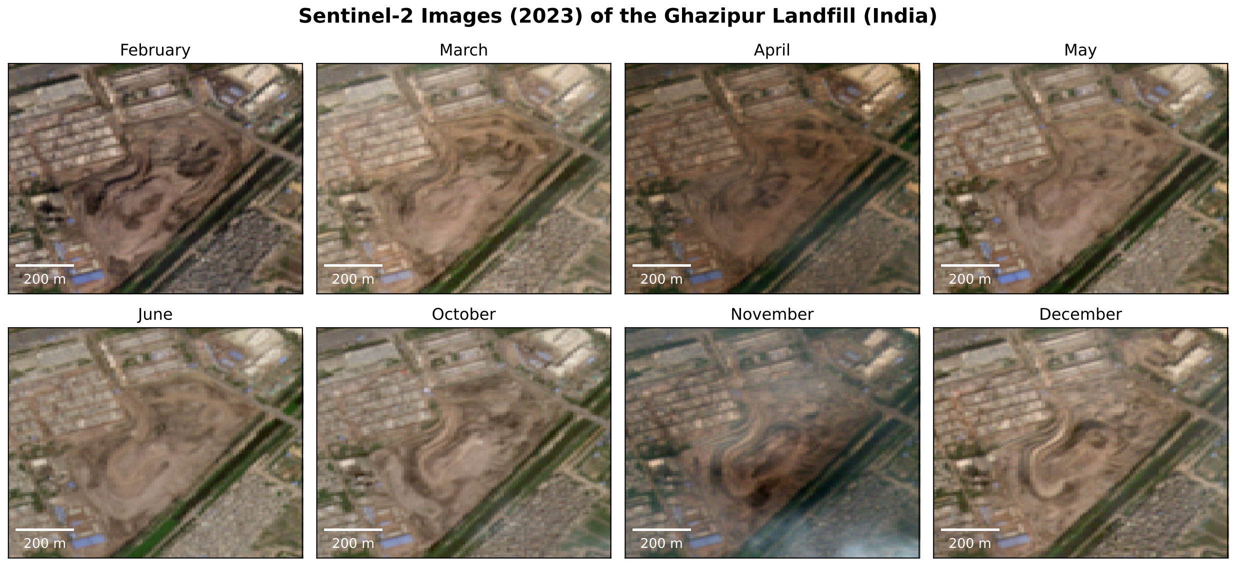

Figure S18: Monthly Sentinel-2 RGB images[12] captured in 2023 showing the Ghazipur Landfill in India. Movie S2 shows a time-lapse sequence of all cloud-free Sentinel-2 RGB images captured throughout 2023.

Table S1: Zero-emission cases: Sites with clear-sky overpasses by EnMAP or EMIT without a detected plume.

Country	Landfill Name	Instrument	Emission (t h^{-1})	Emission (t h^{-1}) excluding non-detection cases	Number of Plumes	Number of Clear-sky Overpasses
India	Bandhwari	EMIT	2.9 ± 1.1	1.9 ± 0.7	2	3
India	Deonar	EnMAP	1.4 ± 0.6	0.7 ± 0.3	1	2
India	Okhla	EMIT	2.3 ± 0.9	1.5 ± 0.6	2	3
Jordan	Al Akaider	EMIT	5.7 ± 2.1	4.9 ± 1.8	6	7
Yemen	Al-Azraqin	EnMAP	1.2 ± 0.4	0.6 ± 0.2	1	2

Table S2: Methane emission rates aggregated by country.

Country	Emission (t h^{-1})	Uncertainty (%)
Argentina	28.1 ± 6.6	23.6
Bangladesh	4.1 ± 1.6	39.8
Brazil	25.6 ± 6.3	24.5
China	10.7 ± 4.4	41.2
India	41.4 ± 5.0	12.1
Iran	11.5 ± 3.2	28.2
Israel	11.4 ± 2.7	24.1
Jordan	11.9 ± 2.7	22.8
Kuwait	7.1 ± 2.1	30.1
Mexico	23.7 ± 5.3	22.3
Pakistan	17.2 ± 4.6	26.6
Saudi Arabia	12.0 ± 3.4	28.5
Spain	7.1 ± 2.5	35.0
Tunisia	5.5 ± 2.0	35.9
United States	7.7 ± 2.2	28.0
Uzbekistan	3.7 ± 1.4	37.1
Yemen	0.6 ± 0.2	33.2

Total of HSI landfill emissions in Table S3 by country. The uncertainties on average emissions for individual landfills within a country are assumed to be independent and are combined in quadrature (square root of the sum of squared uncertainties) to obtain the overall uncertainty for that country.

Table S3: Methane emission rates for HSI measured landfills.

Country	Landfill Name	Latitude	Longitude	Plume Counts	Null Detections	Emission (t h^{-1})	Uncertainty (%)
Argentina	González Catán	-34.7849	-58.6665	5	-	2.8 ± 0.9	34.1
Argentina	Norte III	-34.5272	-58.6259	8	-	22.0 ± 6.4	29.2
Argentina	Piedra Blanca	-31.5198	-64.2354	2	-	3.3 ± 1.3	38.8
Bangladesh	Aminbazar	23.7979	90.2988	1	-	4.1 ± 1.6	39.8
Brazil	Caieiras	-23.3467	-46.772	1	-	14.0 ± 4.8	34.3
Brazil	Pedreira	-23.4037	-46.5608	1	-	11.5 ± 4.0	34.7
China	Ürümqi	44.0384	87.8651	5	-	10.7 ± 4.4	41.2
India	Bandhwari	28.4021	77.1717	4	1	2.4 ± 0.8	34.3
India	Bhalswa	28.7418	77.1565	6	-	2.2 ± 0.8	35.9
India	Deonar	19.0727	72.9285	2	1	2.2 ± 0.9	42.9
India	Ghazipur	28.6237	77.3277	9	-	4.0 ± 1.3	33.7
India	Kachara	18.6589	73.8558	1	-	3.8 ± 1.5	41.0
India	Kanjurmarg	19.1233	72.952	4	-	8.3 ± 2.7	32.2
India	Majura	21.1089	72.8081	3	-	6.9 ± 2.1	30.9
India	Manter Wadi	18.4702	73.9537	1	-	3.7 ± 1.5	39.7
India	Okhla	28.5099	77.2849	7	1	1.9 ± 0.7	35.3
India	Pirana	22.9824	72.569	6	-	6.1 ± 2.1	34.8
Iran	Mashhad	36.2392	59.9882	8	-	3.7 ± 1.6	42.2
Iran	Tehran	35.4585	51.3302	9	-	7.8 ± 2.8	36.5
Israel	Dudaim	31.3217	34.7392	9	-	6.2 ± 2.1	33.7
Israel	Tamar	31.1329	35.2013	10	-	5.2 ± 1.8	34.4
Jordan	Al Akaider	32.5143	36.1101	7	1	3.6 ± 1.3	34.9
Jordan	Ghabawi	31.9302	36.1888	14	-	8.4 ± 2.4	28.9
Kuwait	Kabd	29.1634	47.9138	10	-	7.1 ± 2.1	30.1
Mexico	Relleno Sanitario Bicentenario	19.6512	-99.2788	1	-	2.4 ± 1.0	40.6
Mexico	Relleno Sanitario Peña De Gatos	19.4031	-98.8422	3	-	2.4 ± 1.1	45.0
Mexico	Simeprodeso	25.8712	-100.2993	5	-	5.5 ± 2.2	39.0
Mexico	Tecnosilicatos	19.3241	-98.8033	3	-	11.3 ± 4.5	40.0
Mexico	Zumpango	19.7954	-99.01	1	-	2.1 ± 0.9	44.2
Pakistan	Jam Chakro	25.027	67.0359	1	-	5.2 ± 1.9	35.9
Pakistan	Lakhodair	31.6248	74.4176	2	-	12.0 ± 4.2	34.8
Saudi Arabia	Riyadh	24.6155	46.8953	11	-	12.0 ± 3.4	28.5
Spain	Pinto	40.2636	-3.6316	1	-	7.1 ± 2.5	35.0
Tunisia	Borj Chakir	36.7371	10.0775	5	-	5.5 ± 2.0	35.9
United States	Charlotte Motor Speedway	35.3405	-80.6579	3	-	4.9 ± 2.0	41.8
United States	Seminole Road	33.6621	-84.257	2	-	2.9 ± 0.8	26.5
Uzbekistan	Akhangaran	41.0967	69.4838	8	-	3.7 ± 1.4	37.1
Yemen	Al-Azraqin	15.477	44.1545	1	1	0.6 ± 0.2	33.2

'Null Detections' refers to cases where EnMAP or EMIT has clear-sky overpasses but no plume is detected.

Table S4: Comparison of landfill methane emission rates between HSI estimates and observational estimates (OBS) from previous studies.

Country	Landfill Name	Latitude	Longitude	HSI ($t\ h^{-1}$)	OBS ($t\ h^{-1}$)	HSI Year	OBS Report Year	OBS Source
Argentina	Norte III	-34.5291	-58.6222	22.0 ± 6.4	21.9 ± 7.8	2022, 2023	2021	GHGSat[3]
India	Ghazipur	28.6238	77.3278	4.0 ± 1.3	1.6 ± 1.1	2022, 2023	2021	GHGSat[3]
India	Kanjurmarg	19.1232	72.9535	8.3 ± 2.7	6.4 ± 4.0	2020, 2021, 2023	2021	GHGSat[3]
Iran	Tehran	35.4587	51.33	7.1 ± 2.8	5.0 ± 1.0	2022, 2023	2022	EMIT[13]
Pakistan	Lakhodair	31.6257	74.4179	12.0 ± 4.2	7.1 ± 3.1	2022, 2023	2020	GHGSat[3]
Spain	Pinto	40.259	-3.6357	7.1 ± 2.5	6.6 ± 0.9	2023	2018	In-situ[14]
United States	Charlotte Motor Speedway	35.3393	-80.6585	4.9 ± 2.0	2.9 ± 1.0	2023	2022	AVIRIS-NG[15]
United States	Seminole Road	33.6623	-84.2577	2.9 ± 0.8	2.9 ± 1.1	2023	2022	ASU GAO[15]

Table S5: Comparison of landfill methane emission rates between HSI and the Climate TRACE inventory.

Country	Landfill Name	HSI ($t\ h^{-1}$)	Climate TRACE ($t\ h^{-1}$)	Climate TRACE Report Source	Climate TRACE Report Year
Argentina	González Catán	2.8 ± 0.9	2.2	Waste Atlas	2013
Argentina	Norte III	22.0 ± 6.4	3.3	Waste Atlas	2013
Argentina	Piedra Blanca	3.3 ± 1.3	1.7	METER/OSM	2022
Bangladesh	Aminbazar	4.1 ± 1.6	1.5	METER/OSM	2022
India	Bandhwari	2.4 ± 0.8	0.02	Global Plastic Watch	2021
India	Bhalswa	2.2 ± 0.8	1.4	Waste Atlas	2013
India	Deonar	2.2 ± 0.9	2.4	Waste Atlas	2013
India	Ghazipur	4.0 ± 1.3	2.0	Waste Atlas	2013
India	Kachara	3.8 ± 1.5	0.3	Global Plastic Watch	2021
India	Kanjurmarg	8.3 ± 2.7	0.4	Global Plastic Watch	2021
India	Majura	6.9 ± 2.1	0.2	Global Plastic Watch	2021
India	Manter Wadi	3.7 ± 1.5	0.3	Global Plastic Watch	2021
India	Okhla	1.9 ± 0.7	1.9	METER/OSM	2022
India	Pirana	6.1 ± 2.1	2.2	Waste Atlas	2013
Iran	Tehran	7.8 ± 2.8	20.5	Waste Atlas	2013
Jordan	Al Akaider	3.6 ± 1.3	1.6	Waste Atlas	2013
Jordan	Ghabawi	8.4 ± 2.4	7.3	Waste Atlas	2013
Kuwait	Kabd	7.1 ± 2.1	1.5	METER/OSM	2022
Mexico	Relleno Sanitario Bicentenario	2.4 ± 1.0	1.3	MEX INEGI	2016
Mexico	Simeprodeso	5.5 ± 2.2	17.9	MEX INEGI	2022
Pakistan	Jam Chakro	5.2 ± 1.9	2.0	Waste Atlas	2013
Saudi Arabia	Riyadh	12.0 ± 3.4	1.9	METER/OSM	2022
Spain	Pinto	7.1 ± 2.5	1.6	E-PRTR	2021
United States	Charlotte Motor Speedway	4.9 ± 2.0	0.7	EPA GHGRP	2021
United States	Seminole Road	2.9 ± 0.8	1.4	EPA GHGRP	2021
Yemen	Al-Azraqin	0.6 ± 0.2	1.0	METER/OSM	2022

The "E-PRTR" and "EPA GHGRP" data are directly from the European Pollutant Release and Transfer Register (E-PRTR) and the U.S. Environmental Protection Agency's (EPA) Greenhouse Gas Reporting Program (GHGRP). Other Climate TRACE estimates are based on modeling results.

Table S6: Comparison of HSI emission rates and observational estimates (OBS) from previous studies with Climate TRACE inventory for the top 20 highest emitting landfills from Climate TRACE.

Country	Landfill Name	Latitude	Longitude	Climate TRACE ($t\ h^{-1}$)	HSI ($t\ h^{-1}$)	OBS ($t\ h^{-1}$)	OBS Source
Iran	Tehran	35.4585	51.3302	20.5	7.8 ± 2.8	5.0 ± 1.0	EMIT [13]
Mexico	Simeprodeso	25.8712	-100.2993	17.9	5.5 ± 2.2	-	-
Chile	Loma Los Colorados	-32.957	-70.7962	11.8	10.7 ± 3.9	1.2 ± 0.3	AVIRIS-NG [15]
Mexico	Los Laureles	20.5461	-103.1751	11.8	3.4 ± 1.4	-	-
Greece	Fyli	38.0748	23.6489	10.2	5.3 ± 2.6	-	-
Mexico	Relleno Sanitario	32.4073	-116.7459	9.3	6.9 ± 2.4	-	-
	Portezuelos					-	-
China	West New Territories	22.4193	113.9329	8.6	7.7 ± 2.7	-	-
Mexico	Relleno Sanitario	18.9827	-98.1368	7.8	1.7 ± 0.7	-	-
	Puebla					-	-
Jordan	Ghabawi	31.9302	36.1888	7.3	8.4 ± 2.4	-	-

Table S7: Comparison of landfill methane emission rates estimated using HSI and the city-level WasteMAP inventory.

Country	City	Landfills	WasteMAP ($t\ h^{-1}$)	HSI ($t\ h^{-1}$)	$\frac{HSI}{WasteMAP}$
Argentina	Buenos Aires	Norte III (8), González Catán (5)	3.8	24.8 ± 6.5	6.5
Bangladesh	Dhaka	Aminbazar (1)	3.9	4.1 ± 1.6	1.1
Brazil	São Paulo	Caieiras (1), Pedreira (1)	9.8	25.6 ± 6.3	2.6
Iran	Tehran	Tehran (9)	1.9	7.8 ± 2.8	4.1
Jordan	Amman	Ghabawi (14)	1.1	8.4 ± 2.4	7.6
Kuwait	Kuwait City	Kabd (10)	10.0	7.1 ± 2.1	0.7
Mexico	Mexico City	Zumpango (1), Relleno Sanitario Peña De Gatos (3) Relleno Sanitario Bicentenario (1), Tecnosilicatos (3)	12.5	18.2 ± 4.8	1.5
Mexico	Monterrey	Simeprodeso (5)	0.3	5.5 ± 2.2	16.3
Pakistan	Lahore	Lakhodair (2)	6.0	12.0 ± 4.2	2.0
Pakistan	Karachi	Jam Chakro (1)	5.3	5.2 ± 1.9	1.0
Saudi Arabia	Riyadh	Riyadh (11)	11.8	12.0 ± 3.4	1.0
Spain	Madrid	Pinto (1)	0.3	7.1 ± 2.5	26.8
Tunisia	Tunis	Borj Chakir (5)	0.3	5.5 ± 2.0	18.2
Uzbekistan	Tashkent	Akhangaran (8)	0.9	3.7 ± 1.4	4.0
Yemen	Sanaa	Al-Azraqin (1)	1.6	0.6 ± 0.2	0.4

The HSI emission estimates account for the cumulative methane emissions from individual landfills within each city. There can be additional waste facilities within the city with emissions not observed by the HSI. The numbers in brackets following each landfill name represent the number of detected plumes.

Table S8: Comparison of landfill methane emission rates estimated using HSI and the country-level Climate TRACE inventory.

Country	Climate TRACE (t h^{-1})	HSI (t h^{-1})	$\frac{\text{HSI}}{\text{Climate TRACE}}$ (%)
Argentina	60.3	30.9 ± 6.7	51.3
Bangladesh	24.5	8.2 ± 2.3	33.4
Brazil	247.8	51.1 ± 8.8	20.6
China	681.5	10.7 ± 4.4	1.6
India	108.9	41.4 ± 5.0	38.0
Iran	41.5	19.3 ± 4.3	46.5
Israel	22.4	11.4 ± 2.7	50.8
Jordan	16.0	20.3 ± 3.6	127.0
Kuwait	36.7	14.3 ± 3.0	38.9
Mexico	476.6	47.4 ± 7.5	10.0
Pakistan	55.3	34.5 ± 6.5	62.4
Saudi Arabia	59.4	23.9 ± 4.8	40.3
Spain	52.5	14.3 ± 3.5	27.2
Tunisia	10.3	11.1 ± 2.8	107.2
United States	690.4	7.7 ± 2.2	1.1
Uzbekistan	21.1	7.5 ± 2.0	35.5
Yemen	11.3	1.2 ± 0.3	10.3

The HSI estimation accounts for the cumulative methane emissions from individual landfills within each country. There can be additional landfills within each country with emissions not observed by the HSI analysis presented here.

Movie S1. Time-series of Sentinel-2 RGB images in 2023 for the Ghabawi landfill.

Movie S2. Time-series of Sentinel-2 RGB images in 2023 for the Ghazipur landfill.

References

(1) Varon, D. J.; McKeever, J.; Jervis, D.; Maasakkers, J. D.; Pandey, S.; Houweling, S.; Aben, I.; Scarpelli, T.; Jacob, D. J. Satellite Discovery of Anomalously Large Methane Point Sources From Oil/Gas Production. *Geophys. Res. Lett.* **2019**, *46*, 13507–13516.

(2) Varon, D. J.; Jacob, D. J.; Jervis, D.; McKeever, J. Quantifying Time-Averaged Methane Emissions from Individual Coal Mine Vents with GHGSat-D Satellite Observations. *Environ. Sci. Technol.* **2020**, *54*, 10246–10253.

(3) Maasakkers, J. D.; Varon, D. J.; Elfarsdóttir, A.; McKeever, J.; Jervis, D.; Mahapatra, G.; Pandey, S.; Lorente, A.; Borsdorff, T.; Foorthuis, L. R.; Schuit, B. J.; Tol, P.; van Kempen, T. A.; van Hees, R.; Aben, I. Using Satellites to Uncover Large Methane Emissions from Landfills. *Sci. Adv.* **2022**, *8*, eabn9683.

(4) Varon, D. J.; Jacob, D. J.; McKeever, J.; Jervis, D.; Durak, B. O. A.; Xia, Y.; Huang, Y. Quantifying Methane Point Sources from Fine-Scale Satellite Observations of Atmospheric Methane Plumes. *Atmos. Meas. Tech.* **2018**, *11*, 5673–5686.

(5) Sherwin, E. D.; Rutherford, J. S.; Chen, Y.; Aminfard, S.; Kort, E. A.; Jackson, R. B.; Brandt, A. R. Single-Blind Validation of Space-Based Point-Source Detection and Quantification of Onshore Methane Emissions. *Sci. Rep.* **2023**, *13*, 3836.

(6) Sherwin, E. D.; El Abbadi, S. H.; Burdeau, P. M.; Zhang, Z.; Chen, Z.; Rutherford, J. S.; Chen, Y.; Brandt, A. R. Single-Blind Test of Nine Methane-Sensing Satellite Systems from Three Continents. *Atmos. Meas. Tech.* **2024**, *17*, 765–782.

(7) Esri; Maxar; Geographics, E.; the GIS User Community ESRI World Imagery. 2022; https://www.esri.com/en-us/legal/copyright-proprietary-rights (accessed: 2025-07-08).

(8) Heikenfeld, M.; Marinescu, P. J.; Christensen, M.; Watson-Parris, D.; Senf, F.; van den Heever, S. C.; Stier, P. Tobac 1.2: Towards a Flexible Framework for Tracking and Analysis of Clouds in Diverse Datasets. *Geosci. Model Dev.* **2019**, *12*, 4551–4570.

(9) Zhang, X.; van der A, R.; Ding, J.; Eskes, H.; van Geffen, J.; Yin, Y.; Anema, J.; Vagasky, C.; L. Lapierre, J.; Kuang, X. Spaceborne Observations of Lightning NO_2 in the Arctic. *Environ. Sci. Technol.* **2023**, *57*, 2322–2332.

(10) Jacob, D. J.; Turner, A. J.; Maasakkers, J. D.; Sheng, J.; Sun, K.; Liu, X.; Chance, K.; Aben, I.; McKeever, J.; Frankenberg, C. Satellite Observations of Atmospheric Methane and Their Value for Quantifying Methane Emissions. *Atmos. Chem. Phys.* **2016**, *16*, 14371–14396.

(11) MacLean, J.-P. W.; Girard, M.; Jervis, D.; Marshall, D.; McKeever, J.; Ramier, A.; Strupler, M.; Tarrant, E.; Young, D. Offshore Methane Detection and Quantification from Space Using Sun Glint Measurements with the GHGSat Constellation. *Atmos. Meas. Tech.* **2024**, *17*, 863–874.

(12) Sentinel-2 Cloud-Optimized GeoTIFFs. https://registry.opendata.aws/sentinel-2-l2a-cogs/, 2024.

(13) Thorpe, A. K.; Green, R. O.; Thompson, D. R.; Brodrick, P. G.; Chapman, J. W.; Elder, C. D.; Irakulis-Loitxate, I.; Cusworth, D. H.; Ayasse, A. K.; Duren, R. M.; Frankenberg, C.; Guanter, L.; Worden, J. R.; Dennison, P. E.; Roberts, D. A.; Chadwick, K. D.; Eastwood, M. L.; Fahlen, J. E.; Miller, C. E. Attribution of Individual Methane and Carbon Dioxide Emission Sources Using EMIT Observations from Space. *Sci. Adv.* **2023**, *9*, eadh2391.

(14) Tu, Q.; Hase, F.; Schneider, M.; García, O.; Blumenstock, T.; Borsdorff, T.; Frey, M.; Khosrawi, F.; Lorente, A.; Alberti, C.; Bustos, J. J.; Butz, A.; Carreño, V.; Cuevas, E.; Curcoll, R.; Diekmann, C. J.; Dubravica, D.; Ertl, B.; Estruch, C.; León-Luis, S. F.; Marrero, C.; Morgui, J.-A.; Ramos, R.; Scharun, C.; Schneider, C.; Sepúlveda, E.; Toledano, C.; Torres, C. Quantification of CH_4 Emissions from Waste Disposal Sites near the City of Madrid Using Ground- and Space-Based Observations of COCCON, TROPOMI and IASI. *Atmos. Chem. Phys.* **2022**, *22*, 295–317.

(15) Carbon Mapper data. 2024; Retrieved from https://data.carbonmapper.org, (accessed: 2025-07-08).
