# Supplementary figures and images for: Global Identification of Solid Waste Methane Super Emitters Using Hyperspectral Satellites
Source: Environ Sci Technol. 2025 Aug 19;59(34):18134–45. doi: 10.1021/acs.est.4c14196 (PMC12409875; doi:10.1021/acs.est.4c14196)

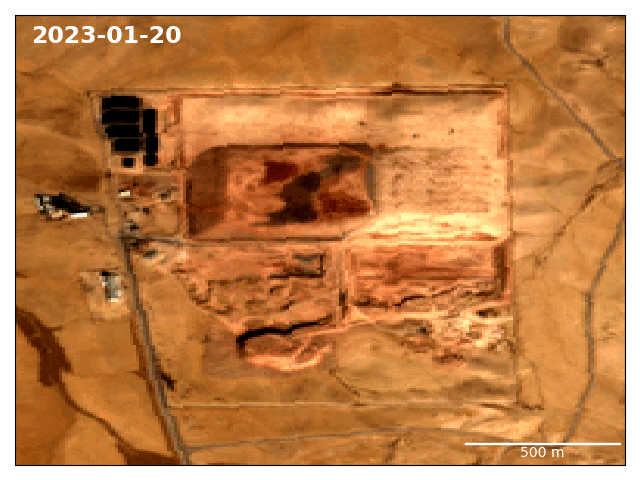

Supplement: Supplementary file 2 [file es4c14196_si_002.zip › Movies/Movie_s1.gif]

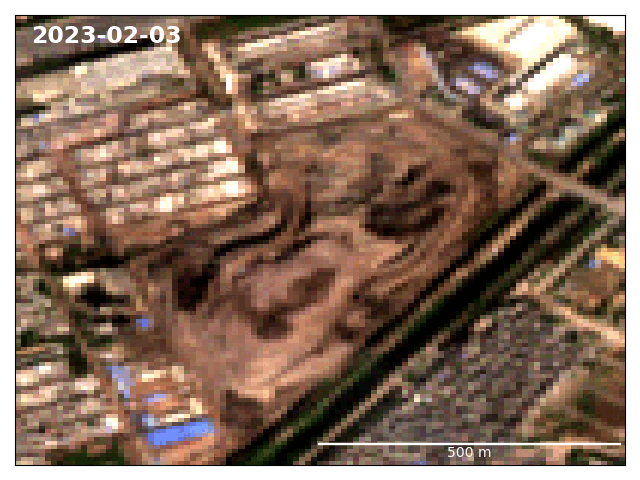

Supplement: Supplementary file 2 [file es4c14196_si_002.zip › Movies/Movie_s2.gif]
